# Structural and Functional Diversity of Animal Toxins Interacting With GPCRs

**DOI:** 10.3389/fmolb.2022.811365

**Published:** 2022-02-07

**Authors:** Anne-Cécile Van Baelen, Philippe Robin, Pascal Kessler, Arhamatoulaye Maïga, Nicolas Gilles, Denis Servent

**Affiliations:** ^1^ CEA, Département Médicaments et Technologies pour La Santé (DMTS), SIMoS, Université Paris-Saclay, Gif-sur-Yvette, France; ^2^ CHU Sainte Justine, Université de Montréal, Montreal, QC, Canada

**Keywords:** toxins, GPCR, structure, peptides, venom

## Abstract

Peptide toxins from venoms have undergone a long evolutionary process allowing host defense or prey capture and making them highly selective and potent for their target. This has resulted in the emergence of a large panel of toxins from a wide diversity of species, with varied structures and multiple associated biological functions. In this way, animal toxins constitute an inexhaustible reservoir of druggable molecules due to their interesting pharmacological properties. One of the most interesting classes of therapeutic targets is the G-protein coupled receptors (GPCRs). GPCRs represent the largest family of membrane receptors in mammals with approximately 800 different members. They are involved in almost all biological functions and are the target of almost 30% of drugs currently on the market. Given the interest of GPCRs in the therapeutic field, the study of toxins that can interact with and modulate their activity with the purpose of drug development is of particular importance. The present review focuses on toxins targeting GPCRs, including peptide-interacting receptors or aminergic receptors, with a particular focus on structural aspects and, when relevant, on potential medical applications. The toxins described here exhibit a great diversity in size, from 10 to 80 amino acids long, in disulfide bridges, from none to five, and belong to a large panel of structural scaffolds. Particular toxin structures developed here include inhibitory cystine knot (ICK), three-finger fold, and Kunitz-type toxins. We summarize current knowledge on the structural and functional diversity of toxins interacting with GPCRs, concerning first the agonist-mimicking toxins that act as endogenous agonists targeting the corresponding receptor, and second the toxins that differ structurally from natural agonists and which display agonist, antagonist, or allosteric properties.

## Introduction

Traditional medicine has always been inspired by nature as a source of care and even today many active components are found in the animal and plant kingdoms (Newman and Cragg, 2016). Venoms of venomous animals constitute a vast library of biochemically stable peptide toxins with particular pharmacological properties, which have evolved to provide their host with capture or defense capabilities. It is estimated that the 200,000 species of venomous animals existing on earth could produce around 40 million toxins, which are still largely unexploited (Gilles and Servent, 2014). Toxins are composed of natural and post-transitionally modified amino acids, including often cysteines, giving them particular cross-linking patterns and various functional three-dimensional structures. Due to a specific evolutionary process which happens in many animal lineages independently, toxins may acquire high affinity and selectivity for their respective targets, explaining their major impact on hemostatic, cardiovascular or central and peripheral nervous systems of prey. In addition, the biological effects and molecular targets of many toxins present in venoms are still unknown, which explains why venom screening to identify new ligand-receptor pairs has gained momentum. Currently, out of the 7,000 toxins discovered and characterized so far, a large majority are associated with the detrimental effect of the venoms via their interaction with voltage-gated or ligand-gated ion channels. Nevertheless, some of them are usefully exploited as insecticides or therapeutic agents, such as ziconotide from *Conus magus* venom, a blocker of the calcium channel Ca_V_2.2 used as a painkiller in morphine-resistant patients (Williams et al., 2008). Interestingly, some therapeutic agents isolated from venoms also target the GPCR superfamily. That is the case of exenatide, the synthetic version of exendin-4 isolated from the saliva of the Gila monster, which is a glucagon-like peptide-1 (GLP-1) analogue which is marketed for the treatment of type 2 diabetes. Given their huge diversity and the multiplicity of unexplored targets, it is likely that many toxin/target combinations with high therapeutic importance are still to be discovered.

GPCRs form the largest family of membrane proteins in mammals with approximately 800 different members, representing more than 30% of therapeutic targets (Rajagopal et al., 2010). They are constituted by seven transmembrane α-helices linked by three external and three internal loops. Once activated by endogenous ligands, these receptors undergo conformational changes allowing the coupling to heterotrimeric G proteins. For example, in class A receptors the transmembrane helices V and VI are moved outward from the center of the receptor, creating a binding site for Gα proteins at the cytoplasmic face of the receptor (Lu et al., 2021). These mechanisms allow the engagement of the receptor in specific intracellular signaling pathways, in order to control almost all physiological processes in humans. Lack of knowledge about the toxin-GPCR interaction highlights the importance of accumulating structural, pharmacological and molecular data on these interactions. Today, little is known about the mechanism of action of these toxins or their structure-activity relationship, and even less about the structure of toxin-GPCR complexes.

In this review, we have classified the GPCR-interacting toxins in two main categories: those that strongly resemble an endogenous ligand, with high structural and biological similarity, called agonist-mimicking peptides, and the other peptides, which are not related to endogenous ligands and display agonist, antagonist or allosteric properties on their respective target GPCRs. The molecular and structural aspects of toxins/receptor interaction leading to diverse modes of action and diverse pharmacological functions will be developed and structure-activity relationships and engineering data of some toxins will be detailed. When relevant, the potential therapeutic applications of toxins are also presented.

## Agonist-Mimicking Toxins

### General Considerations

Toxins present in venoms display a huge diversity of sequences and three-dimensional structures including sometimes a high homology with natural hormones or neurotransmitters produced in non-venomous animals to control several physiological functions. Understanding the evolutionary origin of venom peptides and, in particular, the structural adaptations that underlie their unique biophysical properties is very challenging, even if studies show that 3D structure analysis can be used to identify the evolutionary connections between toxins and their ancestral non-toxic precursors (Undheim et al., 2016). Interestingly, during this recruitment process from endogenous body proteins, toxins may undergo a weaponization process associated with key structural adaptations. This is the case, for example, of spiders and centipede toxins that have evolved from hyperglycemic hormones (Undheim et al., 2015). In parallel, agonist-like peptides present in venoms may also exert a deleterious effect due to their high toxic concentration in prey. This is the case, for example, for cono-insulin, used by net-hunting cone snails to induce hypoglycemic shock in fish (Safavi-Hemami et al., 2015) or for sarafotoxins from Atractaspis snakes, which induce a lethal vasoconstriction in their prey by interacting with endothelin receptors. In the first part of this review, we will focus on six well characterized agonist-mimicking toxin families interacting with GPCRs and isolated from cone snails, snakes and the Gila monster. We will describe their isolation, structure-function analysis, structural characterization and *in vivo* effects, which cover a large diversity of functional effects. Due to limited information and in particular to the lack of structure-function studies, other agonist-mimicking toxins will not be considered. This is the case, for example, of conorfamide toxins, such as the conorfamide-Sr1 isolated from *Conus spurius* (Maillo et al., 2002). This RFamide peptide with an RF sequence at its C-terminal (C-ter) end elicits hyperactivity by the presumed activation of the MrgprC11 receptor, a Mas-related GPCR (Espino et al., 2018). However, many conorfamides are known to target ion channels (Campos-Lira et al., 2017), suggesting that this conotoxin family is not specific to GPCR interaction. Finally, BmK-YA, an enkephalin-like peptide, recently isolated from the scorpion *Buthus martensii* Karsch, appears to be an agonist of mammalian opioid receptors, especially the δ-subtype (Zhang et al., 2012). Unfortunately, nothing is known about the mode of action or structure-activity relationship of this toxin.

### Exendin-4/GLP-1: GLP-1 Receptor

Glucagon-like peptide-1 (GLP-1) is an incretin hormone involved in glucose homeostasis, targeting the GLP-1 receptor (GLP-1R). This receptor is expressed in most cell types and organs, but its most documented effect is an increase in glucose-dependent insulin secretion by pancreatic β-cells (Eng et al., 1990). In these β-cells, GLP-1R also promotes insulin synthesis, proliferation and protection against apoptosis. GLP-1 also has cardiovascular protective and neuroprotective effects, and reduces appetite and food intake. This has made the GLP-1R a therapeutic target of choice for the treatment of type 2 diabetes. This GPCR is coupled to Gαs, provoking a cyclic adenosine monophosphate (cAMP) increase, and β-arrestin recruitment, both of which are drivers of insulin secretion (Montrose-Rafizadeh et al., 1999; Tomas et al., 2020)*.* Unfortunately, the short half-life of GLP-1 (around 2 min) means it is impossible to use it for an anti-diabetic treatment.

The composition and activity of venom produced in the salivary glands of the Gila monster, *Heloderma suspectum*, has been investigated. This has led to the isolation of a 39-amino-acid peptide, designated exendin-4 (Ex-4), showing 53% sequence homology with GLP-1. Exendin-4 targets GLP-1R in pancreatic β-cells (Eng et al., 1992) and shares a similar pharmacological profile with GLP-1 (Raufman et al., 1982). Its sequence was determined by mass spectrometry and sequencing of small peptide fragments obtained by digestion with trypsin (HGEGTFTSDLSKQMEEEAVRLFIEWLKNGGPSSGAPPPS), with the C-ter amidated (Eng et al., 1990). mRNA coding of these toxins recovered from venom glands has been performed (Chen et al., 2006). However, more recently, the proteome of the Gila monster venom was studied by 2D gel electrophoresis and tandem mass spectrometry-based on *de novo* peptide sequencing followed by protein identification based on sequence homology. A total of 39 different new proteins were identified and fill the gaps in the study of toxins from this venom (Sanggaard et al., 2015). ^125^I-exendin-4 (9–39) competition radioligand binding experiments highlighted that exendin-4 and GLP-1 bind to human pancreatic GLP-1R with a similar affinity (IC_50_ = 8.9 and 8.7 nM, respectively) (Mann et al., 2010).

**FIGURE 1:**
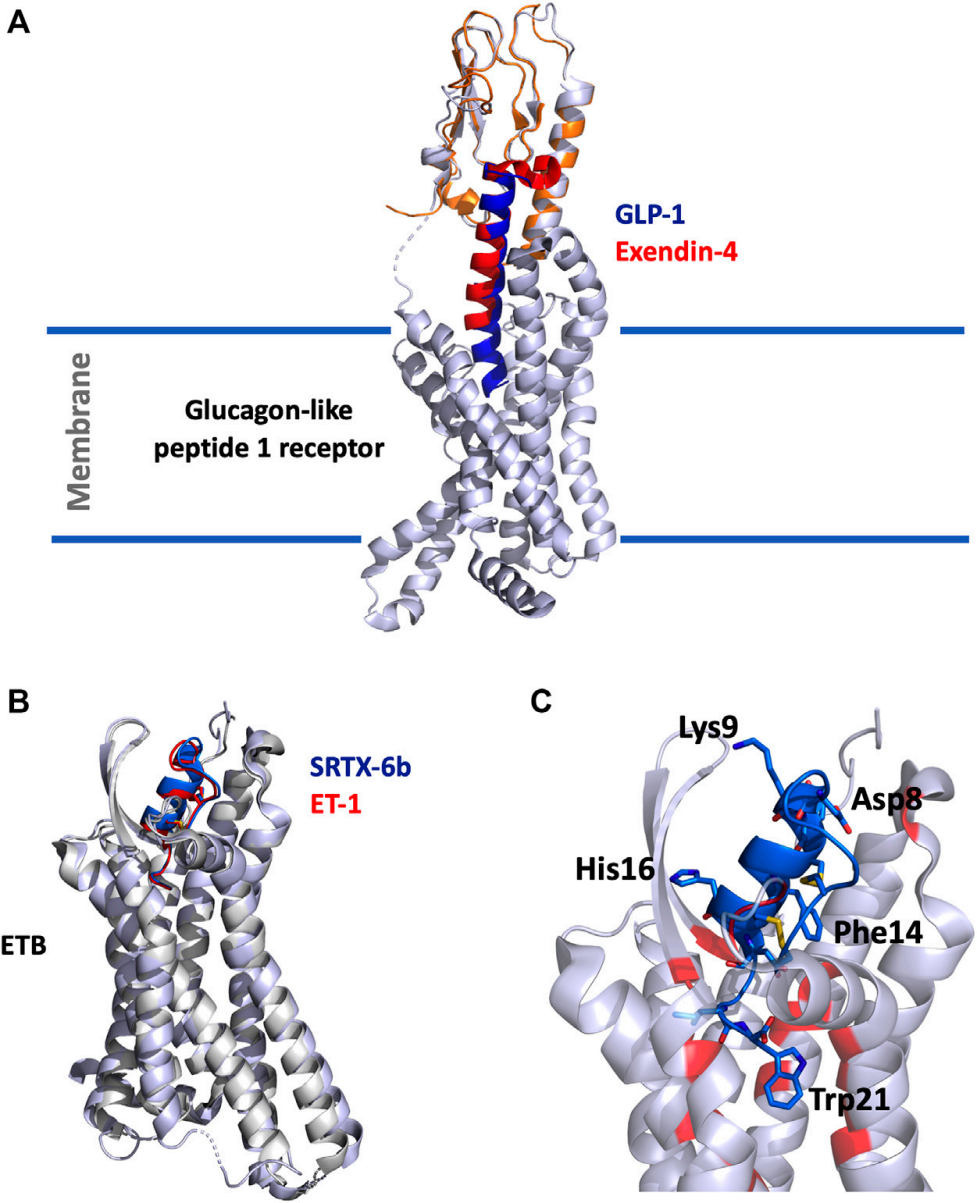
**(A)**, Overlay of the cryo-EM structure of GLP-1 (5vai) with GLP-1R and the crystal structure of exendin-4 (9–33) (3c5t) with the extracellular domain of GLP-1R. The structure is shown in cartoon, light grey for GLP-1R, orange for GLP-1R extracellular domain, dark-blue for GLP-1 and red for exendin-4 (9–33). **(B)**, Superimposition of SRTX-6b (5glh) and ET-1 (6lry) in interaction with ETB. The structures are shown in cartoon, light grey for ETB, blue for SRTX-6b and red for ET-1. **(C)**, Zoom on the interaction site. SRTX-6b is shown in blue, the critical and conserved residues are represented in sticks. The disulfide bonds are shown in yellow. Contact residues on ETB are colored in red.

**FIGURE 2:**
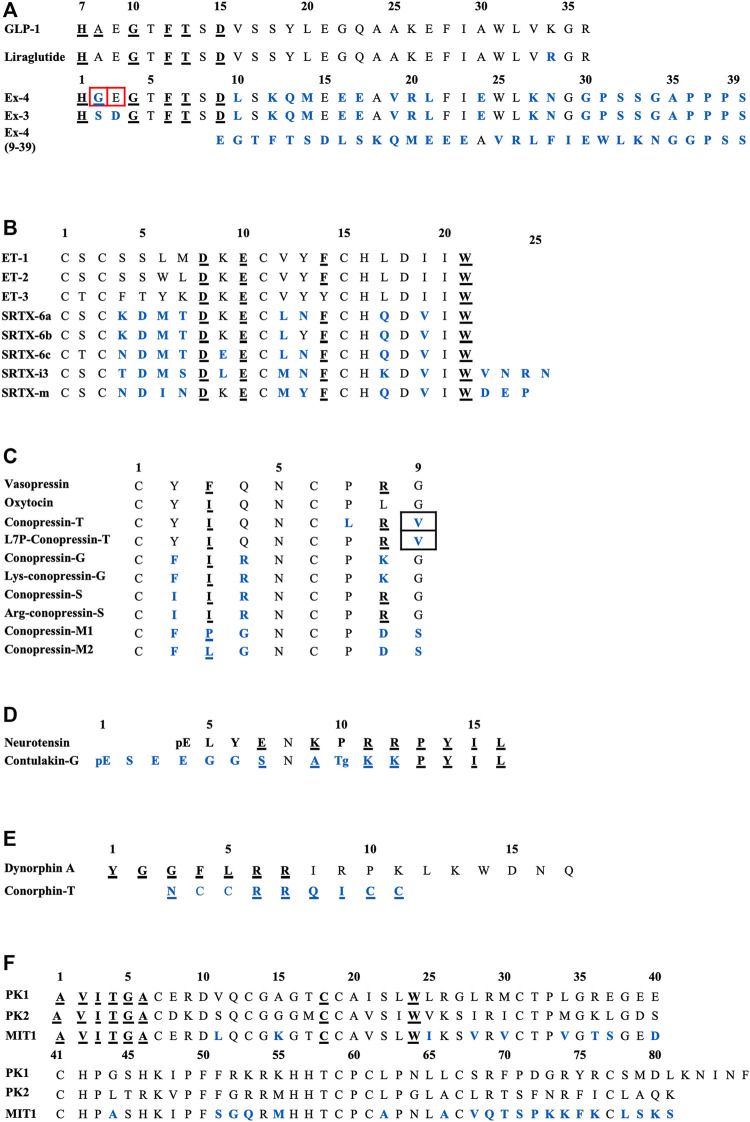
Sequences alignments of agonist-mimicking toxins and homologues. **(A)**, GLP-1, exendin-4. **(B)**, Endothelins and sarafotoxins. **(C)**, Vasopressin, oxytocin and conopressins. **(D)**, Neurotensin and contulakin-G. **(E)**, Dynorphin-A and conorphin-T. **(F)**, Prokineticins and MIT1. In blue, residues which are different from the endogenous ligand; underlined, those critical for affinity/activity; in black boxes, those responsible for the switch towards an antagonist mode of action and in red boxes, those responsible for the switch towards an agonist mode of action. Tg: O-glycosylated-Thr, pE: pyroglutamate.

The structure of the GLP-1 in complex with GLP-1R and its associated G-protein Gαs was solved recently by cryo-electron microscopy (cryo-EM) (Zhang et al., 2017) (Figure 1A). In addition, the crystal structure of the N-terminal (N-ter) ecto-domain of GLP-1R in complex with the truncated peptide exendin-4 (9–39) was also solved at 2.2 Å resolution (Runge et al., 2008) (Figure 1A), as was the crystal structure of GLP-1 complexed with the extracellular domain of GLP-1R (Underwood et al., 2010). The hydrophobic binding site of GLP-1R is defined by discontinuous segments including primarily a well-defined α-helix in the N-ter of GLP-1R and a loop between two antiparallel β-strands (Runge et al., 2008). Exendin-4 forms a single helix between residues 11–27 and has a Glu residue (Glu16) at the position equivalent to Gly22 in GLP-1 (Figure 2A) (Neidigh et al., 2001). However, the first six residues of GLP-1 are not really structured, leading to the formation of two helices from positions 13–20 and 24–35 linked around Gly22 (Thornton and Gorenstein, 1994). The C-ter regions of exendin-4 and GLP-1 interact the same way with the N-ter extracellular domain of the GLP-1R. This allows the interaction of the N-ter part of exendin-4 with the transmembrane domain of the receptor leading to its activation (Zhang et al., 2015). Conformational changes are then facilitated, regulated by the G protein and other intracellular partners. As highlighted in Figure 2A, GLP-1 and exendin-4 contain a similar N-ter sequence necessary for activity on GLP-1R (Liang et al., 2018). Furthermore, binding assays have demonstrated that the N-ter positions 7, 10, 12, 13, and 15 of GLP-1 are important for affinity at GLP-1R (Suzuki et al., 1989; Adelhorst et al., 1994) and that the first histidine of GLP-1 at position 7 as a free N-ter amino acid is very important in stimulating insulin release. The same amino acids are present in exendin-4, confirming their critical role in the binding to GLP-1R (Suzuki et al., 1989), as confirmed in the 3D structure of the complex. Surprisingly, exendin-4 has a higher affinity for the N-ter extracellular domain of GLP-1R (nGLP-1R) than GLP-1 (IC_50_ = 6 and 1,120 nM, respectively) (Runge et al., 2007). This difference may be explained either by non-conserved residues in the central part of the two ligands or by the specific C-ter extension of exendin-4. Interestingly, the role of this C-ter extension (PSSGAPPPS) in the binding of exendin-4 to GLP-1R was excluded (Runge et al., 2008), reinforcing the critical importance of the Leu10-Gly30 sequence of exendin-4 in its high affinity for nGLP-1R.

The switch from an agonist to antagonist mode of action is highly dependent on a few amino acid substitutions. Indeed, exendin-3 (Ex-3) is an analogue of exendin-4, isolated from the Mexican beaded lizard *Heloderma horridum*. It differs from exendin-4 by two substitutions, Ser2-Asp3 in place of Gly2-Glu3, making their bioactivities completely different: exendin-3 being an antagonist of GLP-1R (Eng et al., 1992; Chen et al., 2006). The truncated form of exendin-4 (9–39) is also a competitive antagonist of GLP-1R. It has a high affinity for GLP-1R (IC_50_ = 0.6 nM) and is able to inhibit GLP-1 and exendin-4 from binding to the receptor. Many analogue peptides have been synthesized in order to understand the determinants that enable exendin-4 (9–39) to have antagonist activity, whereas the truncated GLP-1 (15–36), for instance, remains an agonist (Patterson et al., 2011). Glu16, Val19, and Arg20 were shown to be the essential determinants of exendin-4 (9–39)’s antagonism (Runge et al., 2008). Moreover, the removal of the first two N-ter residues of GLP-1 yielded a partial agonist with a 100-fold reduction in affinity (Montrose-Rafizadeh et al., 1999). Interestingly, exendin-4 has a Gly8 in place of an Ala8 and an additional C-ter extension of nine amino acids which is absent from GLP-1, preventing degradation of the peptide from dipeptidyl peptidase IV (DPP IV) and neutral endopeptidase, and enhancing its half-life compared with GLP-1 (Doyle et al., 2003). It should be noted that a biased agonist of GLP-1R called P5 has also been discovered, promoting G-protein signaling comparable to GLP-1 and exendin-4, but exhibited a significantly reduced β-arrestin response. It shares a common C-ter sequence with exendin-4, but only 4% homology with the N-ter sequence, which interacts with GLP-1R, explaining this biased activity (Zhang et al., 2015; Liang et al., 2018).

Thus, like GLP-1, on isolated rat islets, exendin-4 inhibits glucagon secretion (Silvestre et al., 2003), stimulates insulin synthesis (Alarcon et al., 2006), and protects against β-cell apoptosis in response to different insults (Ferdaoussi et al., 2008). In type 2 diabetes patients, exendin-4 decreases blood glucose and raises β-cell sensitivity to glucose when given twice daily subcutaneously for 1 month (Egan et al., 2003). After subcutaneous administration, exenatide (exendin-4 synthetic form) reaches a maximum plasma concentration in 2 h, and the mean half-life is almost 4 h. Due to its longer half-life (almost 120 times higher), exenatide was the first anti-diabetic drug GLP-1-based therapy to reach the market, in 2005 (Furman, 2012). Today, seven drugs derived from GLP-1 have been approved, with the idea of improving the half-life to space out the medication (Madsbad, 2016). With the idea of improving the half-life of exenatide as a therapeutic tool, some efforts have been made with pharmacomodulations: exenatide LAR (long-acting release) is formulated with exenatide in microspheres commonly used in extended drug release formulations. Once weekly subcutaneous injection is thought to be the desired frequency, and it is currently undergoing phase III clinical trials (Weill Medical College of Cornell University, 2017). On the other hand, liraglutide is a GLP-1 analog with two modifications: a substitution of Arg34 for Lys34 and an attachment of a C-16 free fatty acid derivative via a glutamoyl spacer to Lys26, known to delay the absorption rate by 13 h (Knudsen et al., 2000), allowing a once daily injection which prevents the progression of diabetic nephropathy. So far, many “glutide” drugs have been approved and marketed (Nauck et al., 2021).

### Sarafotoxin/Endothelin: Endothelin Receptors ETA/ETB

Endothelins (ET1, ET2, and ET3), initially described as endogenous regulators of the cardiovascular system, mediate their effects through two class A GPCRs, named ETA and ETB. These receptors are widely expressed and are coupled to the Gαq, Gαi/o, Gα12/13 G-protein pathways. Among the multiple effects of endothelins, the most documented is cardiovascular and participates in vascular tone control at the level of endothelial and vascular smooth muscle cells. In the vasculature, the vascular smooth muscle cells express both ETA and ETB receptors, while endothelial cells express only ETB. Activation of ETB in endothelial cells causes vasorelaxation through the generation of nitric oxide, while activation of ETA and/or ETB in smooth muscle cells induces vasoconstriction (Davenport et al., 2016).

The bolus intravenous injection of either nonselective or ETB-selective agonists in rats results in an initial and transient vasorelaxation followed by a long-lasting vasoconstriction and associated elevation of mean arterial pressure (Betts and Kozlowski, 2000). ET-1 is considered as the most potent endogenous vasoconstrictor (Yanagisawa et al., 1988), but also induces contraction of many other smooth muscles such as airway, uterine and prostate smooth muscles (Kozuka et al., 1989; Maggi et al., 1989; Kobayashi et al., 1994). Besides their contractile action, ET receptors have mitogenic and anti-apoptotic effects in various cell types. The ETB receptor is also involved in the development of the neural crest during embryonic development and ETB mutations are associated with megacolon formation characteristic of Hirschsprung disease (Hosoda et al., 1994; Lahav et al., 1996). The ETB receptor is also described as a clearance receptor allowing elimination of circulating ET-1 in the lung (Fukuroda et al., 1994). Several cardiovascular and renal diseases are associated with the endothelin system, in particular, pulmonary arterial hypertension for which endothelin receptor antagonists ambrisentan (Volibris^®^) and bosentan (Tracleer^®^) are used for therapeutic treatment (Enevoldsen et al., 2020). Endothelin receptors have also been shown to be overexpressed in various cancers, such as ovarian carcinoma, melanoma, prostate, lung, renal, and colon cancers (Nelson et al., 2003; Rosanò et al., 2013).

Sarafotoxins (SRTXs) form a family of toxins isolated from the venom of *Atractaspis engaddensis* (burrowing asp) and other Atractiaspidae, venomous snakes from the Middle East. SRTXs are among the most toxic snake toxins with lethal dose of 0.15 μg/kg body weight in mice (Ducancel, 2005). They exert their toxic effect by inducing a strong general vasoconstriction leading to heart failure. The first sarafotoxins, SRTX-6a, -6b, and -6c, were discovered by fractionation of the venom by HPLC and analysis of the cardiotoxicity of each fraction in mice (Kloog et al., 1988; Takasaki et al., 1988). These three peptides were found to be highly homologous in sequence and structure between them and also with the endothelin family of peptides. Like endothelins, they are twenty-one amino acids long and contain two conserved disulfide bridges, making them bicyclic peptides. More recently, the long SRTX-m and SRTX-i3 families, containing two to ten amino acid extensions at their C-ter, were discovered (Hayashi et al., 2004).

Given their high homology with endothelin peptides, SRTXs mediate their effects through the endothelin receptors and behave as endothelin mimicking peptides. The ETA and ETB receptors differ in their properties in binding to endothelins and SRTXs. The ETA receptor is selective and binds the three endothelins with the selectivity order ET-1>ET-2>>ET-3. ETA binds ET-1 with an affinity in the sub-nanomolar range, and ET3 with and affinity at least 1000-fold lower. By contrast, the ETB receptor is not selective and binds the three endothelin peptides as well as SRTX-6a, 6b and 6c, with affinities in the sub-nanomolar range. SRTX-6b binds ETA with nanomolar affinity, whereas SRTX-6c exhibits only micromolar affinity for ETA, making SRTX-6c an ETB-selective ligand (Ducancel, 2005; Barton and Yanagisawa, 2019). In contrast to short SRTX, long SRTX-m and SRTX-i3 only show moderate affinity for ETB (K_D_ > 300 nM) and virtually do not bind to ETA (K_D_ > 50 µM) (Mourier et al., 2012). However, truncation of their additional C-ter tail at position 21 drastically increases their affinity and makes them as potent as SRTX-6b on ETB. Surprisingly, long SRTX-m, SRTX-I3 and short SRTX-6b have different hemodynamic and respiratory effects (Mahjoub et al., 2015, 2017; Malaquin et al., 2016).

The sequence alignment of SRTX and endothelins (Figure 2B) reveals the high homology of these peptides and shows the conserved residues. Among them are the four cysteine residues organized in a unique motif Cys1-X-Cys3—Cys11-X-X-X-Cys15 and forming Cys1-Cys15 and Cys3-Cys11 disulfide bridges. The other conserved residues are Asp8, Lys9 (except for SRTX-6c), Glu10, Phe14 (except for ET-3). The C-ter part of the peptide is the most conserved with invariant residues His16, Asp18, Ile20, and Trp21. By contrast the sequence at positions 4-7 represents the variable region of the ET and SRTX peptide family. The structure of these peptides obtained by NMR shows that they contain an extended region (residues 1–4), followed by a turn (residues 5–7) and an α-helicoidal part (residues 8–15). The extended region, connected to the helical region by the two disulfide bridges, constitutes a cysteine stabilized domain, while the C-ter part of the peptide (16–21) is conformationally variable and can adopt multiple conformations. This structural organization is conserved in both short and long SFTX (Mourier et al., 2012).

Structure-activity relationship studies have revealed the crucial determinants for endothelin and SRTX binding and activity. Early studies demonstrated that substitution or modifications of the invariant Trp21 resulted in a loss of function of ET-1 in pulmonary artery contraction assays. Comparable results were obtained by substitution of the other invariant residues Asp8, Glu10, and Phe14. The effect of ET-1 was also abolished by the reduction of the four cysteines or their replacement by alanines (Nakajima et al., 1989; Tam et al., 1994).

In the last 5 years, several crystallographic structures of the ETB receptor complexed with various ligands have been published (Shihoya et al., 2016, 2018; Izume et al., 2020). The structures of ETB complexed with ET-1 or with SRTX-6b reveal that the positions of the two peptides in the receptor are virtually identical (Figure 1B). For both peptides, the C-ter part dives into the hydrophobic pocket of the orthosteric site of the receptor located in its core heptahelical domain. The placement of the C-ter Trp21 residue at the bottom of the binding site is consistent with the previous observation that longer peptides, i.e. long SRTX or un-matured endothelins, do not bind to ET receptors with good affinity. The other part of the peptide, the cysteine stabilized helical domain, is sandwiched between the second extracellular loop (ECL) and the extracellular ends of the transmembrane helices VI and VII. The structures also revealed that the conserved residues of the ET/SRTX family are all involved in the interaction with the receptor. Moreover, Trp21, which has been experimentally shown to be crucial for binding and activation of the receptor, interacts with the tryptophan residue of the CWXP motif involved in the signaling function of class A GPCRs (Figure 1C).

Considering the importance of the pathophysiological role of the endothelin system, many ligands targeting the endothelin receptors have been developed for therapeutic purposes. Most of them are antagonists with various selectivity profiles. Despite the number of clinical studies done with peptide and non-peptide antagonists, only bosentan and ambrisentan, small molecules with a slight selectivity towards ETA, have proved their efficacy and are on the market for the treatment of pulmonary arterial hypertension (Davenport et al., 2016). In the face of this unappealing clinical picture, the unique indication for which an endothelin agonist has emerged with a positive outcome is stroke treatment. Indeed, IRL-1620 (Sovateltide), currently in phase III clinical trials, has yielded better recovery from acute cerebral ischemic stroke (Gulati et al., 2021; Pharmazz, Inc. 2021). IRL-1620 is a linear, truncated, modifier ET/SRTX family peptide that acts as a selective and potent ETB agonist. IRL-1620 is a 14-amino-acid peptide, corresponding to amino acids 8–21 of ET-1 in which Cys11 and Cys15 have been replaced by alanine, Lys9 by glutamic acid, as in SRTX-6c, and the amino-terminal end is succinylated. This peptide, acting as an ETB agonist, enhances neurogenesis, angiogenesis and protects neural cells from apoptosis in rats (Leonard and Gulati, 2013; Briyal et al., 2019).

### Conopressins/Oxytocin-Vasopressin: Oxytocin/Vasopressin Receptor

The oxytocin/vasopressin signaling system constitutes one of the most complex and important neuroendocrine systems in humans. Oxytocin (OT) and arginine-vasopressin (AVP) mediate their biological effects by acting on specific receptors. AVP mediates its actions through three known vasopressin receptors: V1aR, V1bR, and V2R. V1a receptors are expressed in the liver, vascular smooth muscle cells, brain and in many other tissues. In the vasculature, V1aR mediate the pressor actions of AVP by a phospholipase C-mediated pathway. In the brain, V1aR mediates the anxiety-producing responses to AVP (Ring, 2005). V1b receptors, present in the anterior pituitary, mediate the adrenocorticotrophic hormone-releasing effects of AVP. V1bR is also expressed in the brain, the kidney and the adrenal medulla. Recently, V1bR has been shown to mediate anxiety and stress in rats and in humans (Ishizuka et al., 2010). V2 receptors, present in the collecting duct of the kidney, mediate the antidiuretic action of AVP by an adenylate cyclase-mediated pathway. The AVP pathways of V1aR-mediated vasoconstriction and V2R-induced water retention represent a potentially attractive target of therapy for edematous diseases. Experimental and clinical evidence suggests beneficial effects of AVP receptor antagonists by increasing free water excretion and serum sodium levels. Thus, the incidence of cardiovascular diseases may be enhanced by a dysregulation of OT and AVP levels (Szczepanska-Sadowska et al., 2021). New AVP receptor antagonists have been developed in order to treat chronic heart failure, liver cirrhosis and syndrome of inappropriate antidiuretic hormone secretion (Ali et al., 2007). OT mediates its actions through OT receptors expressed in the uterus, mammary gland, ovary, brain, kidney, heart, bone, and endothelial cells (Gimpl and Fahrenholz, 2001). In the uterus, OT receptors mediate the uterine contracting effect of OT. The OTR is capable of binding to either Gαi or Gαq proteins and activates a set of signaling cascades, such as the MAPK, PKC, PLC, and CaMK pathways (Devost et al., 2008). The central effects of OT continue to be the focus of intense investigative scrutiny in animals and in humans (Meyer-Lindenberg et al., 2011), as a possible therapeutic agent for the treatment of autism and other anxiety disorders.

Conopressin toxins isolated from *Conus* venoms constitute a wide family of peptides, known to target the vasopressin/oxytocin receptor family. Among them, conotoxins Ba1, Ba2, and Ba3 have been isolated from *Conus bayani* by using transcriptomics and mass sequencing (Rajaian Pushpabai et al., 2021). Above all, conopressin-T (cono-T) was isolated from the venom of *Conus tulipa* by its ability to induce a scratching effect after injection in mice. Its sequence was determined by *de novo* mass spectrometry sequencing: CYIQNCLRV. Cono-T belongs to the vasopressin-like peptide family and displays high sequence homology with the mammalian hormones oxytocin and vasopressin (Dutertre et al., 2008). Dutt et al. conducted the first venomics approach on venom’s ducts of *Conus tulipa* by integrating transcriptomics and proteomics: they identified several conotoxin precursors across two specimens of *Conus tulipa*, notably concerning conopressins. From the proteome, a mass corresponding to conopressin-T (1,107.6 Da) was identified. However, due to its anatomic distribution in the duct, conopressin-T may play a role in defense despite a suggested predatory role. That might explain the antagonist effect on vasopressin receptors (Dutt et al., 2019a). Indeed, cono-T is a selective V1aR antagonist (K_i_ = 319 nM), has a partial agonist activity at the oxytocin receptor (K_i_ = 108 nM), producing 22% of AVP and OT maximal activity, a partial activity on V1bR, producing 9% of AVP and OT maximal activity, and no detectable activity on V2 receptors. In CHO cells expressing V1aR, cono-T induces inhibition of AVP-stimulated IP production. Finally, cono-T does not stimulate phospholipase C activity in cells expressing V1aR (Dutertre et al., 2008). Conopressins of the vasopressin family are all characterized by a disulfide-containing ring between residues 1–6 and a short exocyclic C-ter tripeptide between residues 7–9. As highlighted in Figure 2C, residues 7 and 9 are conserved in OT and AVP (Pro7 and Gly9), whereas they are replaced by Leu7 and Val9 in conopressin-T (Giribaldi et al., 2020). Conopressins, AVP and OT share the same structural loop between the two cysteine residues, but the C-ter fragment of cono-T is not superimposed with the tail region of AVP or OT (Dutertre et al., 2008). The agonist binding site is mainly made by the three extracellular domains of the oxytocin receptor, whereas the different binding sites of various antagonist ligands are formed by transmembrane helices 1, 2, and 7 (Postina et al., 1996; Giribaldi et al., 2020). It should be noted that Arg8 is essential for pressor activity and enables a tight interaction with V2R. An aromatic residue at position 3 decreases, the potency at all other receptors, but enhances peptide selectivity for V1aR and V1bR. Finally, the presence at position 4 of a basic residue instead of a glutamine diminishes the potency at all tested receptors, especially at V2R (Giribaldi et al., 2020).

Many other peptides belonging to the family of conopressins have been isolated from other species and have distinct pharmacological profiles with human receptors*.* Among them, conopressin-G (cono-G) from *Conus geographus* venom and conopressin-S (cono-S) from *Conus striatus* venom were the first conopressins identified (Cruz et al., 1987). They both cause severe itching and scratching in mice within a few minutes after injection. Although the sequences of these conopressins are close to that of vasopressin itself, they have an additional positive charge in position 4. Cono-S has a similar affinity for OTR, but is less potent at V1aR and does not bind to V2R. Cono-S binds with high affinity to V1bR (K_i_ = 8.3 nM) (Dutertre et al., 2008)*.* Lys-conopressin-G, an analogue of cono-G (Nielsen et al., 1994), and Arg-conopressin-S, an analogue of cono-S, are characterized by an amidated C-ter tail and two basic amino acids: one Arg in position 4 and a Arg or Lys in position 8, outside of the cyclic structure (Cruz et al., 1987).

Recently, Giribaldi et al., discovered and characterized two new conopressins from the venom gland transcriptome of *Conus miliaris*, called conopressin-M1 (cono-M1) and conopressin-M2 (cono-M2). It was highlighted that the amidated form of cono-M1 has a partial agonist activity at V1bR and at V1aR for both the amidated and acid forms. Conopressin-M2 is a full agonist at the V2R, albeit with low micromolar affinity. Thus, the low activity of cono-M1 and cono-M2 can be explained by the substitution of Gln4 by a glycine residue, the absence of a basic residue in position 8 and the missing glycine residue in position 9 (Giribaldi et al., 2020). L7P-cono-T is a more potent analogue of cono-T on V1aR, but has a similar affinity for OTR. Like cono-T, it induces inhibition of IP production in CHO without stimulating phospholipase C activity in cells expressing V1aR. L7P-cono-T differs from AVT only at position 9 and acts as a V1aR antagonist. This modification alone can switch peptide activity from agonist to antagonist (Dutertre et al., 2008). The biological effects in animals are not yet well known. As AVP, it is likely that conopressins have a role in renal homeostasis, for instance.

### Contulakin-G/Neurotensin: Neurotensin Receptors

Neurotensin (NT), a hypotensive peptide, first isolated from bovine hypothalamus, acts as a neurotransmitter and neurotransmodulator in the central nervous system (CNS), and also as a local hormone in the small intestine (Carraway and Leeman, 1973). This tridecapeptide is an agonist of neurotensin receptors (NTSRs) with a sub-nanomolar affinity (Checler et al., 1986). Three of them, NTSR1 and NTSR2, which are both GPCRs, and NTSR3, a single transmembrane domain sorting receptor, have been identified as targets (Vincent et al., 1999). Neurotensin is involved in many central biological processes, but also in many peripheral effects like gastrointestinal motility and vasodilatation (Carraway and Leeman, 1973). In the CNS, NT exerts various effects, including analgesia (Clineschmidt and McGuffin, 1977) and central control of blood pressure (Rioux et al., 1981), is involved in the pathophysiology of schizophrenia and Parkinson’s disease, with levels of endogenous NT and NTSR1 expression decreased in patients with the symptoms of schizophrenia (Nemeroff, 1980) (Clineschmidt and McGuffin 1977; Yamada et al., 1995; St-Gelais et al., 2006). In the gastrointestinal tract, NT has effects on pancreatic endocrine secretion and colonic motility, and decreases gastric acid secretion (Osumi et al., 1978). NT has a high affinity for NTSR1 and activates PLC through the Gαq/11-coupled pathway, producing inositol triphosphate (IP3) and DAG diacylglycerol (DAG). This pathway induces activation of PKC and mobilization of intracellular calcium, key oncogenic effectors (Chabry et al., 1994). NTSR2, like NTSR1, is present in both the CNS and peripheral organs. It is coupled to the Gαq/11-dependent PLC signal pathway, Gαi/o and Gα12/13 (Sarret et al., 1998; Vincent et al., 1999). Finally, NTSR3 forms a heterodimer with NTSR1 on the surface of HT29 cells, activating the IP3/PKC signaling pathway (Martin et al., 2002). NT is particularly involved in the occurrence of gastrointestinal cancers with increased levels of NT and NTSR1 (Clineschmidt and McGuffin, 1977; Nemeroff, 1980; Yamada et al., 1995; St-Gelais et al., 2006). NT and NTSR1 are then promising candidates for clinical screening for gastrointestinal cancers because of their overexpression in these cancers, and are promising targets because the inhibition of NTSR1 expression or the knockdown of the *NTS1* gene decreases oncotic MMP-9 expression and activity (Dong et al., 2017; Sánchez and Coveñas, 2021). Finally, NT has central opioid-independent anti-nociceptive effects, leading to improved therapeutic management of pain (Chartier et al., 2021).

By testing fractions from the venom of *Conus geograp*hus on the loss of motor control in mice, a peptide named contulakin-G has been isolated by HPLC. It is a linear 16-amino-acid peptide whose sequence was determined by Edman sequencing and *O*-glycosidase and β-galactosidase hydrolysis: pESEEGGSNATKKPYIL, with the O-glycosylation [β-D-Gal*p*-(1→3)-α-D-Gal*p*NAc-(1→) on Thr10.

By measuring phosphoinositide accumulation in CHO cells expressing NTSR1, NTSR2, and NTSR3, Craig et al. determined the agonist potency of synthetic contulakin-G. This toxin is an agonist for all three subtypes of neurotensin receptors with sub-micromolar potency (IC_50_ = 0.96, 0.73 and 0.25 μM, respectively) (Craig et al., 1999). Contulakin-G is less potent than NT, but shares a common C-ter sequence (KKPYIL) which is highly conserved and responsible for their interaction with the receptor and their biological activities (Figure 2D). The substitution of Glu7 of NT by a positive or non-charged amino acid decreases the desensitization potency and the substitution of Lys9 decreases the agonist potency, thus explaining the lower potency of contulakin-G. Finally, electrostatic interactions seem very important: replacing the two conserved Arg residues diminishes the agonist potency (Lee et al., 2015). SAR studies for contulakin-G highlight the importance of the glycoamino acid in determining interactions with neurotensin receptors. It has been demonstrated that removing this glycosylated residue resulted in an increase of the affinity for all the receptor subtypes, with an IC_50_ of contulakin-G for NTSR1 40 times higher than the IC_50_ of deglycosylated contulakin-G (Craig et al., 1999). Interestingly, contulakin-G exhibits higher potency *in vivo*: after intracerebroventricular (icv) injection in mice of both forms, contulakin-G induces loss of motor control at lower doses than the deglycosylated form (Craig et al., 1999). This is probably due to the slowdown of proteolytic degradation thanks to the O-glycan. To support the role of glycosylation of contulakin-G, some molecular docking simulations have been performed on both the contulakin-G forms in interaction with NTSR1. The glycosylation of Thr10 modifies the conformation of contulakin-G in comparison with the deglycosylated form. Indeed, the salt bridges between the terminal carboxyl of the peptide and Arg94 and Arg241 of NTSR1 facilitate interactions. The Tyr145 residue, which is near these two Arg, favors the stabilization of these residues by cation-pi interactions. Thus, this anchoring point formed by the salt bridge between the C-ter of the peptide and the receptor leads to the repositioning of the Lys12 so that it can form a hydrogen bond with Glu332. When the peptide is glycosylated, this major interaction between Lys12-Glu332 is lost (Lee et al., 2015). Thus, the desensitization of neurotensin receptors is enhanced by the presence of both glycosylation of contulakin-G and charged amino acid residues.

In *in vivo* experiments, beagles were injected for many days with infusions or bolus doses of contulakin-G, leading to biexponential disposition function. The kinetics show a rapid initial redistribution phase followed by a slow terminal elimination phase. It appears that contulakin-G might be metabolized within the CNS or rapidly metabolized in the systemic circulation or bound by tissues (Kern et al., 2007). In the rat, contulakin-G provokes, like neurotensin, central effects as loss of motor control, absence of preening/grooming, reduced sensitivity to tail depression and peripheral effects as gut contraction (Craig et al., 1999). With regard to these interesting biological effects of the toxin, Cognetix developed a synthetic form of contulakin-G, CGX-1160, which in 2005 obtained orphan drug status for the treatment of chronic intractable pain following intrathecal administration in patients with spinal cord injury by the US Food and Drug Administration (FDA). After a break in development, a phase Ia trial was started to determine the safety of escalating doses in patients with central neuropathic pain (Sang et al., 2016).

### Conorphin-T/Dynorphin A: κ-opioid Receptor

The Kappa opioid receptor (KOR) is a GPCR known to modulate the effects of several neurotransmitters such as dopamine and serotonin, and glutamate release in the central nervous system. KOR and its endogenous ligand dynorphin-A (dynA (1–17), with a K_i_ of 0.28 nM) (Goldstein et al., 1981), have a widespread distribution, especially in the CNS, but also in the peripheral system. Dynorphin-A is a neurotransmitter peptide processed from its precursor prodynorphin (Chavkin et al., 1982). It interacts with KOR which is coupled to Gαi/o, leading to inhibition of adenylyl cyclase and a decrease in the production of cAMP, thus modulating the conductance of Ca^2+^ and K^+^ channels. Furthermore, this activation implies kinase cascades including GRK and members of the MAPK family (ERK1/2, p38 and JNK) (Bruchas and Chavkin, 2010). Dysregulation of the dynorphin/KOR system is involved in several psychiatric diseases, including schizophrenia, depression, bipolar disorder, drug addiction, and especially analgesia, thus being an interesting new therapeutic target to investigate as a potential analgesic (Zhang et al., 2007; Koob and Volkow, 2010; Clark, 2020; Sapio et al., 2020). Initially, agonists were thought to treat analgesia: bremazocine was synthesized in an effort to produce opiates with greater KOR selectivity and with minimal morphine-like side effects (Römer et al., 1980), and asimadoline succeeded in phase II for acute attacks of pain in irritable bowel syndrome (Williams and Mangel, 2010; Camilleri, 2011). Finally, KOR antagonists have already reached the market, such as buprenorphin and naloxone to treat mood disorders and morphine addiction.

Extraction of total RNA from the ducts of *Conus textile* and cDNA cloning and sequencing have led to the identification of the cDNA sequence of conorphin-T (NCCRRQICC), a nonapeptide present in *Conus textile* (Luo et al., 2006). The screening of a conopeptide library on KOR highlighted that conorphin-T is a new selective agonist of the KOR that mimics dynorphin-A (Brust et al., 2016). Conorphin-T is a member of the T-superfamily of conotoxins which includes 1,000 distinct active peptides found in the venom of various *Conus* species (Aguilar et al., 2006).

Conorphin-T contains two Arg residues side by side, a spacer amino acid followed by a hydrophobic residue and two Cys on the C-ter tail. Docking studies have shown that even if conorphin-T and dynA have low sequence similarity, the toxin interacts similarly with KOR and the truncated dynorphin-A (1–8) (Brust et al., 2016). Indeed, it was demonstrated that the last nine amino acids at the C-ter of dynA have little impact on KOR activity. However, the N-ter YGGF sequence of dynA is common to endogenous opioid ligands, such as endorphins and enkephalins, and is known to play a major role in opiate receptor affinity. It then appears that the essential pharmacophore of dynA lies within the N-ter fragment YGGFLRRI. The presence of two arginines side by side is often a marker of KOR selectivity (Brust et al., 2016).

Interestingly, the three isomers (beads, globular and ribbon) of conorphin-T were synthesized and radioligand binding assays highlighted that the bead form was the most active one on KOR (K_i_ = 80 nM), compared to the ribbon form (K_i_ = 580 nM) and the globular one (K_i_ = 1,5 µM). Many analogues derived from conorphin-T were also synthesized in order to increase affinity and stability. So, the substitution of Asn1 by Tyr1 (such as dynA) increased affinity for KOR twofold, meaning that an aromatic residue at the N-ter position is important for the affinity. The alanine scan of the peptide also highlighted that the residues 4–7 (RRQI) are important for the affinity on KOR. Furthermore, the presence of an aromatic amino acid at position 6 improved affinity. Thus, the authors identified an active sequence in the C-ter tail: RRQICC, with the two vicinal Cys in C-ter deeply critical for high KOR affinity (Figure 2E). Finally, the glutamine residue is a spacer necessary to the presentation of the peptide to the receptor (Brust et al., 2016).

Like dynA, conorphin-T may have antinociceptive properties. Furthermore, conorphin-1, an analogue peptide of conorphin-T, was recently developed and shown to activate KOR with sub-nanomolar potency and high selectivity above other opioid receptors. In rodent models of nociceptive pain (formalin-induced pain), the analgesic and antiallodynic effects of conorphin-1 were evaluated. However, if the peptide was delivered peripherally by intraplantar injection, no analgesic effect was observed, maybe due to the impossibility of targeting KOR in peripheral sensory nerve endings innervating the skin (Deuis et al., 2015).

### MIT1/Prokineticin/EG-VEGF: Prokineticin Receptors PKR1/PKR2

Prokineticin 1, is an angiogenic factor also known as endocrine gland-derived vascular endothelial growth factor (EG-VEGF) (LeCouter et al., 2001; Li et al., 2001). Prokineticin 2 is a related peptide of prokineticin 1 (87% homology), known to be a mammalian homologue of the frog skin peptide Bv8 (Wechselberger et al., 1999). Both peptides bind to prokineticin 1 and 2 receptors (PKR1 and PKR2), members of the neuropeptide Y receptor family, with nanomolar affinity (Lin et al., 2002; Masuda et al., 2002; Negri et al., 2007). PKR1 is highly expressed in spleen and gastrointestinal tissues, where its activation enhances smooth muscle contraction (Li et al., 2001), whereas PKR2 is widely expressed in the CNS (Masuda et al., 2002). The PKRs are Gαq-coupled receptors and promote intracellular Ca^2+^ mobilization*.* They also may couple to Gαi and Gαs and some other G-proteins, like Gα12 or G13 (Lin et al., 2002; Soga et al., 2002). This activation is known to increase intracellular Ca^2+^ concentration with nanomolar potency in CHO cells (Masuda et al., 2002)*.* Finally, prokineticins and their receptors are widely distributed, which may suggest their role, among others, in angiogenesis in endocrine glands, heart failure, colorectal cancer, etc. (Masuda et al., 2002). So far, antagonists, especially of PKR1, have been developed (Levit et al., 2011).

In 1990, Schweitz et al. identified almost thirty peptides from the venom of *Dendroaspis polylepis*, half of them having a contractile effect on intestinal smooth muscle (Schweitz et al., 1990). In this venom a mamba intestinal toxin-1 (MIT1) was isolated, whose sequence of 81 amino acids and 10 cysteines at identical positions of Bv8 was determined by Edman degradation (AVITGACERDLQCGKGTCCAVSLWIKSVRVCTPVGTSGEDCHPASHKIPFSGQRMHHTCPCAPNLACVQTSPKKFKCLSKS) (Schweitz et al., 1999). MIT1 is a homologue of the human endogenous ligand EG-VEGF and prokineticin 1, sharing 80% sequence identity with them and 58% sequence identity with prokineticin 2 (Li et al., 2001). This toxin is more active and affine for its receptor than EG-VEGF/prokineticin 1 and prokineticin 2, with a K_i_ of 4.1 nM for PKR1 and 0.67 nM for PKR2 (Masuda et al., 2002). The N-ter sequence AVITGA is characteristic of the “AVIT” family of toxins. They are composed of 80–90 amino acids, analogues of MIT1, and are presumed to have a potent effect on intestinal contractility and to increase hyperalgesia (Wen et al., 2005).

The solution structure of MIT1 has been investigated using 2D homonuclear NMR. MIT1 contains ten cysteines, involved or not in disulfide bridges and leading to various disulfide bridging configurations. This may confer a particular importance on this topological information in order to determine accurately the 3D structure. MIT1 is an analogue of colipase, a co-enzyme required for optimal activity of pancreatic lipase, which shares the same cysteine configuration. Several charged residues are buried inside the molecule, whereas some hydrophobic residues, such as Trp24, are exposed on the surface (Boisbouvier et al., 1998). There is little information regarding the SAR of MIT1, but some prokineticin residues conserved in MIT1 have been highlighted in Figure 2F as critical for activity. The N-ter part of AVITGA is highly conserved among prokineticins and analogue proteins and is critical for activity. The deletion of this part leads to inactivation of the peptide. The C-ter rich in cysteine is also essential for bioactivity of prokineticins. The substitution of Cys18 provokes a decrease in activity. Interestingly, the substitution of Ala1 by Met or an addition of Met in the N-ter tail leads to a switch towards an antagonist mode of action (Bullock et al., 2004).

The first identified biological action of MIT1 was the contraction of the isolated guinea pig ileum and distal colon and relaxation of the proximal colon (Schweitz et al., 1999). Interestingly, Bv8, a 77-amino-acid peptide, was isolated from skin secretions of *Bombina* v*ariegata* and *Bombina bombina.* This peptide was also found to stimulate the contraction of gastrointestinal smooth muscle with high potency (Mollay et al., 1999; Negri et al., 2007).

## Toxins Targeting GPCRs

### Toxins With 2 Disulfide Bonds

Toxins with two disulfide bridges are much less present in venoms as compared to toxins with three or four disulfide bridges (Reynaud et al., 2020), and most of them are found in conus venoms. Exceptionally, they were identified in arthropods (Smith et al., 2011; Daly and Wilson, 2018) as disulfide directed β-hairpin (DDH: C-C-C-C framework with the I-III, II-IV connectivity), a fold which seems to be evolutionarily linked to three-disulfide-bridge ICK toxins (Undheim et al., 2016). Interestingly, the same cysteine pattern was also found in apamin, a bee-venom toxin that selectively blocks the small conductance of Ca^2+^-dependent K^+^ channels in CNS (Lazdunski, 1983). There is a large diversity of two-disulfide-bridge toxins in cone snails venoms and they cover five different cysteine frameworks (I, V, X, XIV, and XXIV (Kaas et al., 2012)). The pattern CC-C-C is the most common one, including the α-conotoxins active on nicotinic acetylcholine receptors, the ρ-conotoxins active on adrenoceptors and the χ-conotoxins active on norepinephrine transporter. The CC-CC pattern contains many members belonging to the T-superfamily, including the τ-conotoxins interacting with somatostatin receptors (Petrel et al., 2013). Moreover, the C-CC-C pattern was also recently found in *Conus vexillum* venom, delineating a new αB-conotoxin superfamily (Luo et al., 2013). Finally, the pattern C-C-CC (L-superfamily) and the pattern C-C-C-C (Q superfamily) are patterns of conotoxins for which no clear biological activities have yet been described. Three cysteine frameworks called globular (cysteine I-III; II-IV pattern), ribbon (cysteine I-IV; II-III pattern) and beads (cysteine I-II; III-IV pattern) are compatible with the structure of toxins with 4 cysteines. The globular organization is the most common one including for example the α-, ρ-, and τ-conotoxins. Much fewer conotoxins are organized with the ribbon structure like the χ-conotoxins and no conotoxin with bead organization has yet been described.

### ρ-TIA: Adrenoceptors

Adrenoceptors constitute a family of receptors sensitive to epinephrine and norepinephrine and are divided into three families (α1_A_Rs, α2_A_Rs, and βARs), each of them comprising three members (Hein, 2006). The three α1-adrenoceptors (α1_A_AR, α1_B_AR, and α1_D_AR) are widely expressed in the body and are involved in the control of smooth muscle tone, like vessels, bladder or prostate muscles. They display their actions mostly by the Gαq-pathway. The α1ARs antagonists were developed to treat bladder outlet obstruction in benign prostatic hyperplasia. The last generation of drugs (like tamsulosin), which are more selective for the α1_A_AR subtype, induces less hypotension due to the blockage of the α1_B_AR (Tammela, 1997). The α2ARs (α2_A_AR, α2_B_AR, and α2_C_AR) are presynaptic receptors linked to the Gαi/o pathway. They display various functions like the control of vessel tone, regulation of the sympathetic nervous system or pain transmission. Due to their wide expression, they are practically not used as therapeutic targets (Flordellis et al., 2004). The βARs (β1AR, β2AR, and β3AR) have crucial functions. β1AR is predominantly found in the heart, kidney, and fat cells, and β1AR antagonists are largely used against hypertension (Alhayek and Preuss, 2021). β2AR is mainly expressed in pulmonary cells where its activation by agonists fights against asthma and chronic obstructive pulmonary diseases (Abosamak and Shahin, 2021). β3AR is involved in metabolic effects in adipocytes and in other functions that still need to be better characterized (Dessy and Balligand, 2010).

**FIGURE 3:**
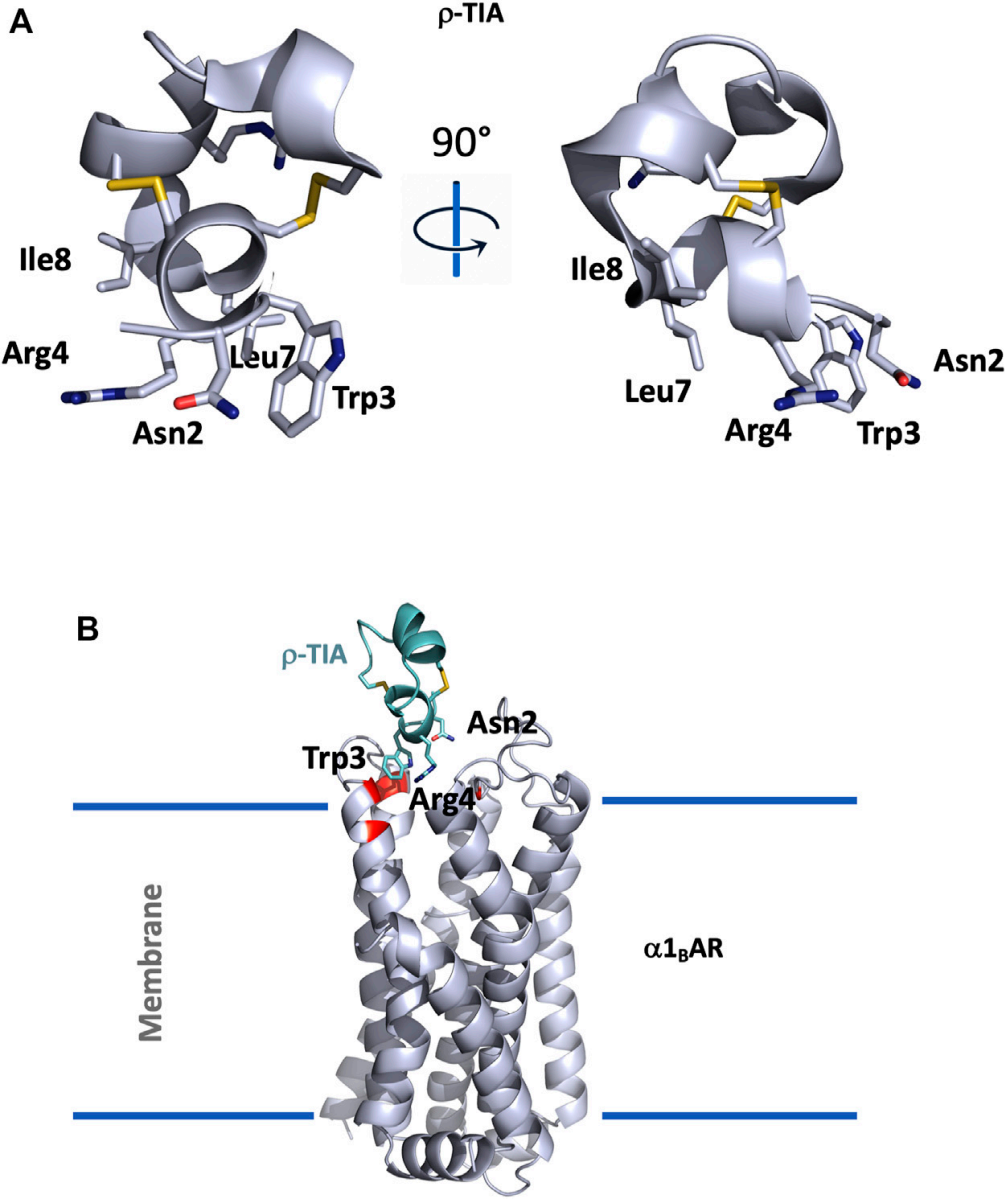
**(A)**, Structural view of ρ-TIA (1ien). ρ-TIA is represented twice in cartoon with a 90° angle rotation; important residues are shown in sticks, disulfide bonds are represented in yellow. **(B)**, Model of α1_B_AR in interaction with ρ-TIA (Ragnarsson et al., 2013). α1_B_AR is represented in light grey cartoon and ρ-TIA in green, important residues for the interaction in the receptor are shown in red.

Two toxin families were discovered acting on adrenoceptors, one conus toxin with two disulfide bonds (the ρ-TIA) and several three-finger-fold toxins, what we call aminergic toxins, as developed in Section 5. ρ-TIA was discovered by a bioguided strategy in the venom of the *Conus tulipa* snail by following the biphasic contractile response of the electrically stimulated rat prostatic vas deferens (Sharpe et al., 2001). ρ-TIA binds to α1 adrenoceptor subtypes.ρ-TIA binds α1_A_AR, α1_B_AR and α1_D_AR with an IC_50_ of 150 μM, 70 and 315 μM, respectively (Sharpe et al., 2001). On its principal target, α1_B_AR (Figure 3B), ρ-TIA is considered as a non-competitive competitor, as saturation binding studies revealed that 1 µM of the toxin reduced maximum ^125^I-HEAT binding by 85% without affecting the affinity of the receptor for the radioligand. Association and dissociation kinetic analysis of the radioligand ^3^H-prazosin in the presence and absence of ρ-TIA suggests also that the toxin acts as a non-competitive ligand. The antagonist property of ρ-TIA was tested in HEK293 cells stably expressing α1ARs with norepinephrine as agonist. On α1_A_AR and α1_D_AR, ρ-TIA shifted to the right the norepinephrine activation curves without affecting its efficacy, which is compatible with a pure competitive effect. On α1_B_AR, ρ-TIA decreases norepinephrine efficacy and acts as an insurmountable antagonist.

ρ-TIA, composed of 19 residues (FNWRCCLIPACRRNHKKFC*) reticulated by two disulfide bridges in a ribbon organization, consists of a stretch of 3.10 helix between Arg4 to Leu8, a helical turn from Pro9 to Arg12, followed by four nested β-turns between Arg12 and Cys19, which almost comprise another turn of helix. These comprise a type I β-turn between residues 12–15, and three type IV β-turns between residues 13–16, 14–17, and 15–18 (Sharpe et al., 2001) (Figure 3A).

An alanine scanning exploration revealed that the N-ter part (Asn2 to Ile8) and Arg12 are involved in the binding with α1_B_AR (Chen et al., 2004). On the receptor side, fourteen receptor residues of the extracellular loops influence ρ-TIA affinity (Ragnarsson et al., 2013). Double mutant cycle analysis and docking analysis point to a close proximity between Arg4 of ρ-TIA and Asp327 and Phe330; Trp3 of ρ-TIA and Phe330 and Ser318; Asn2 of ρ-TIA and Val197 and finally the positive charge of the N-ter part of the toxin and Glu186 of α1_B_AR (Figure 3B). The ρ-TIA binding site described the first allosteric site for α1_B_AR (Ragnarsson et al., 2013). It is not so trivial to understand the exact biological role of toxins, especially when they are described as non-toxic, like most of the toxins targeting GPCRs. *Conus tulipa* has developed a net hunting strategy to catch fish. Targeted fish are immobilized by secretion of so-called “nirvana cabal” peptides to hamper their escape. The tulipa snail can then open its mouth to directly capture fish. Lewis’s team demonstrated that ρ-TIA produced a striking loss of zebrafish larvae escape response to light mechanical touch on their trunk or tail, in a dose-dependent manner, with an EC_50_ around 200 nM (Dutt et al., 2019b). This study highlights the importance of using ecologically relevant animal behavior models to decipher the biological role of animal toxins. In rat, ρ-TIA antagonizes the contractions induced by noradrenaline in the aorta and vas deferens through α1_A_AR and α1_D_AR (Lima et al., 2005). ρ-TIA is then the first α-type toxin active on a GPCR, providing a new function for this peptide family. In addition, this toxin describes a novel allosteric site on α1_B_AR.

### τ-CnVa: Somatostatin Receptor SST3

The discovery of τ-CnVa resulted from the venom gland transcriptome analysis of the cone snail *Conus consors*. The peptide of fourteen amino acids containing two pairs of adjacent cysteine residues (ECCHRQLLCCLRFV) belongs to the Τ-family of conotoxins. τ-CnVa was chemically synthesized and a directed bridging strategy was used to form disulfide bridges in a cysteine I-III, II-IV connectivity pattern, and the C-ter of the peptide was amidated, according to the conserved features of τ-conotoxins. τ-CnVa activity was searched for over a large panel of molecular targets, including GPCRs, voltage-gated ion channels, nicotinic receptors and neurotransmitter transporters, and only the SST3 receptor emerged from this screen. This receptor is a member of the GPCR family of somatostatin receptors comprising five members, whose function is to inhibit the release of hormones such as growth hormone, TSH, pancreatic and gastrointestinal hormones. By means of binding experiments with iodinated somatostatin, τ-CnVa was shown to bind the SST3 receptor with an affinity of 1.5 µM and had an at least fifty-fold lower affinity for the four other somatostatin receptors (SST1, SST2, SST4, and SST5). In functional assays (Ca^2+^ mobilization assay), τ-CnVa was unable to activate the SST3 receptor, but dose-dependently inhibited the somatostatin response with an IC_50_ of 16.8 µM. The mode of interaction with the SST3 receptor and the *in vivo* effect of the toxin have not yet been documented (Petrel et al., 2013).

## Toxins With 3 Disulfide Bonds

Three-disulfide-bond toxins are widely expressed in almost all venomous animals. Nine cysteine patterns of three-disulfide-bond toxins have already been described only in conus snail venoms such as the CC-C-C-CC framework of the µ-conotoxins acting on Na_V_ channels or the CC-C-C-C-C pattern of αA-conotoxins interacting with nAChRs (Kaas et al., 2012). Two frameworks are particularly well known and are represented by hundreds of toxins. The inhibitor cystine knot (ICK) is characterized by the -C-C-CC-C-C- pattern associated with cysteine I-IV; II-V; III-VI connectivity. ICK toxins (23–35 amino acids) are found in many venomous animals, such as spiders, scorpions, cone snails, and sea anemones, and are mainly associated with inhibition of voltage-gated ionic channels (Na_V_, Ca_V_, K_V_, KCa, TRP, and ASIC). The CSαB-related toxins are characterized by the -C-C-C-C-C-C- pattern associated with the classic ICK cysteine I-IV; II-V; III-VI pattern. This toxin class is largely found in scorpions and is called short-scorpion toxin active on voltage-gated potassium channels. Another multifunctional fold including three-disulfide bonds is the Kunitz fold, characterized by peptides 55–65 amino acids long and a -C-C-C-C-C-C- framework with I-VI; II-IV; III-V cysteine connectivity. This structure is known to support mainly anti-protease activity, such as in the BPTI, and potassium channel inhibition (Peigneur et al., 2011). Nevertheless, Kunitz toxins interacting with ASIC, TRPV1 or Ca_V_ channels have already been described, as well as with vasopressin V2R, as will be described below. Until recently, GPCR toxins came from reptiles (mostly snakes) and cone snails. In this chapter, we describe three toxins, one from snakes targeting the vasopressin receptor V2R and two from arachnids targeting melanocortin receptors.

### Mambaquaretin: Vasopressin Receptor V2

The vasopressin receptor 2 (V2R) is essentially expressed in the distal part of the nephron and in the collecting tubule of the kidneys. It regulates water homeostasis under the control of arginine-vasopressin (AVP). Once activated in the collecting duct, the V2R/Gαs pathway stimulates intracellular cAMP production, which activates protein kinase A to phosphorylate aquaporin 2, allowing its translocation from intracellular vesicles to the apical membrane via an intracellular calcium-dependent exocytosis mechanism. Further, water can go through aquaporin 2 at the apical membrane from the urine to the main cell before reaching the blood thanks to aquaporins 3 and 4 at the basolateral membrane (Szczepanska-Sadowska et al., 2021). Both loss- and gain-of-function variants of V2R are associated with human diseases and over 260 mutations have been reported to date (HGMD, 2021.) and recently reviewed (Makita et al., 2020). Despite the primordial function of the V2R and its numerous associated diseases, only two drugs are on the market, DdAVP, a specific V2R agonist, and tolvaptan, a specific V2R antagonist. DdAVP is mainly used to treat insipidus diabetes and tolvaptan to treat hyponatremia and autosomal dominant polycystic disease (Garona et al., 2020).

Mambaquaretin-1 (MQ1) was discovered by a bioguided strategy in the venom of the green mamba *Dendroaspis angusticeps* by competition of the ^3^H-AVP on membrane preparations of COS cells stably expressing hV2R (Ciolek et al., 2017). MQ1 displays low nanomolar affinity for V2R with an absolute selectivity. Indeed, at 1 µM no activity could be found on 156 GPCRs, including the three other AVP-sensitive receptors V1aR, V1bR, and OTR (Ciolek et al., 2017). Because MQ1 adopts a Kunitz-type peptide structure known to inhibit serine proteases and K_V_ channels, it was tested on 45 serine proteases and 9 Kv channels with no activity. Finally, this safety profile was completed by testing fifteen ionic channels (including the eight involved in cardiac activity) (Ciolek et al., 2017). Here again, no activity could be observed. By functional tests, MQ1 displays a pure antagonist effect against AVP (Ciolek et al., 2017). In addition, MQ1 acts as an inverse agonist on the V2R/Gαs pathway (Droctové et al., 2020).

**FIGURE 4:**
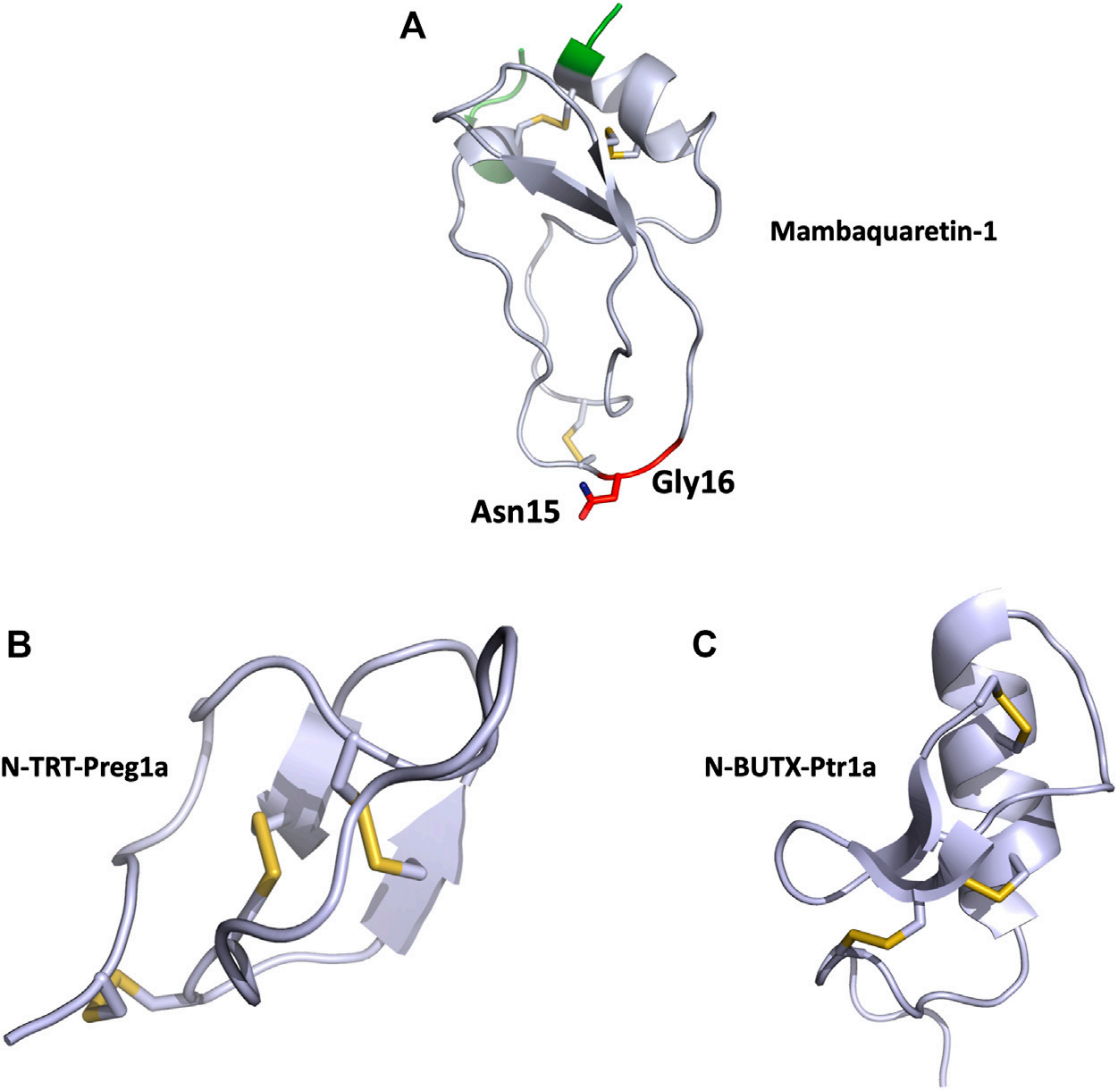
**(A)**, Structural view of mambaquaretin-1 (5m4v). Mambaquaretin-1 is represented in cartoon. In green, residues, which can be deleted without affecting the Kd of the toxin for V2R, critical residues for binding are shown in red, disulfide bonds are shown in yellow. **(B)**, Structural view of N-TRT-Preg1a (6saa) and **(C)**, N-BUTX-Ptr1a (6sab). N-TRT-Preg1a and N-BUTX-Ptr1a are represented in cartoon, disulfide bonds are shown in yellow.

MQ1 is composed of 57 residues (RPSFCNLPVKPGPCNGFFSAFYYSQKTNKCHSFTYGGCKGN ANRFSTIEKCRRTCVG) reticulated by three disulfide bridges, and the structure of the MQ1-N15K-G16A variant was solved by X-ray crystallography (PDB: 5M4V), demonstrating its Kunitz structure (Figure 4A). The two main functions of Kunitz toxins are inhibition of the serine protease mainly through its loop 1 and more particularly through the dyad Lys15-Ala16 in MQ1 and K^+^ channel blockage, mainly by its N-ter part. These two regions were mutated in MQ1, revealing that this toxin binds V2R with the same strategy as BPTI uses to inhibit serine protease (Ciolek et al., 2017).

When V2R is inactive or blocked by an antagonist, urine is no longer concentrated. Diuresis increases while urine osmolality decreases simultaneously. This aquaretic effect was seen in mice (Ciolek et al., 2017) and rats when animals were intraperitoneally injected with various doses of MQ1 (Droctové et al., 2020). MQ1 biodistribution was followed in mice by positron emission tomography (PET) imaging. 24 h post injection, only the kidney presents radioactivity, demonstrating the exclusive biodistribution of MQ1 in this organ. This result is in accordance with the fact that V2R is almost exclusively expressed in the kidney and that MQ1 is the most selective V2R ligand ever discovered (Droctové et al., 2020).

Deficiency in AVP secretion induces hyponatremia with plasma sodium levels below 135 mmol/L in humans. Chronic heart failure, liver failure or chronic kidney disease are associated with hyponatremia related to an increased risk of death. The efficacy of MQ1 was demonstrated in a rat model of hyponatremia and may be of great help in emergencies.

Polycystic kidney diseases (PKDs) are genetic disorders causing end-stage renal failure in children and adults. The inhibition of V2R is still currently the only validated therapeutic strategy in humans to reduce disease progression. MQ1 was validated against this disease in a mouse model. The CD1-pcy/pcy mouse strain suffers from type 3 nephronophthisis, which is similar in many respects to autosomal dominant polycystic kidney disease, due to the mutation T1841G in the gene orthologous to human NPHP3 (Nagao et al., 2012). These pcy mice were given daily I.P. injections of with 13 µg of MQ1 for 100 days. Treated mice presented better renal function and fewer cysts as compared to control ones, demonstrating the *in vivo* efficacy of MQ1 against this chronic disease (Ciolek et al., 2017).

MQ1 is the first Kunitz-type toxin active on a GPCR, providing a new function for this peptide family. Leaving its N- and C-ter extremities far from the V2R interface, these areas are useful for grafting contrast elements for *in vitro* and *in vivo* imaging. MQ1 is the most selective V2R antagonist and the only efficient *in vivo* imaging tool. These two qualities may be exploited to develop diagnostic tools and therapeutics.

### N-TRTX-Preg1a and N-BUTX-Ptr1a: Melanocortin Receptors

The melanocortin (MC) system consists of agonists, antagonists and receptors that control the physiological corticoadrenal functions. The five (MC1–5R) subtypes are regulated by the agonist melanocyte-stimulating hormones (MSH) and the adrenocorticotropin hormones, but also by the endogenous antagonists, agouti and agouti-related proteins. They mostly display their functions by the Gαs/cAMP signal transduction pathway, thus regulating a set of physiological functions including obesity, inflammation, sexual function, pigmentation, cardiovascular tone and steroidogenesis (Dores et al., 2016; Shen et al., 2017; Toda et al., 2017; Fatima et al., 2021). MC3R and MC4R are expressed primarily in the brain. Through binding with the endogenous melanocortin ligands, these receptors play a key role in the regulation of energy homeostasis. For instance, anorexigenic hormones or neurotransmitters such as leptin, insulin, and serotonin activate MC4R in order to reduce food intake and/or increase energy expenditure. On the other hand, in the fasting state, the agonist MSH is down-regulated while the endogenous antagonists are up-regulated, thus promoting feeding (Cone, 2006; Toda et al., 2017).

Venoms are difficult to exploit due to the large number of toxins that composed them. In addition, 90% of venomous animals are tiny and never produce enough venom for the classical bioguided screening strategy. A “venomics” approach combining transcriptomic and proteomic characterization of 191 species identified 20,206 venom toxin sequences. 3,597 toxins were produced by recombinant expression or by chemical synthesis (Gilles and Servent, 2014). Screened on membrane preparations of COS cells stably expressing the human melanocortin receptor 4 (hMC4R) by competition with ^125^I-NDP-α-MSH, this bank gave an incredible hit rate of 8%. Two toxins isolated from this screening have been studied in depth, the spider N-TRTX-Preg1a toxin, exhibiting an inhibitory cystine knot (ICK) motif, and N-BUTX-Ptr1a, a short scorpion toxin with a CSαβ structure (Reynaud et al., 2020).

Both N-TRTX-Preg1a and N-BUTX-Ptr1a display low micromolar affinities for the four MCRs: MC1R, MC3R, MC4R, and MC5R. Due to the ability of ICK and CSαβ scaffolds to bind to ionic channels, the two selected toxins were tested at 100 μM on eight sodium channels, fourteen potassium channels, one voltage-gated calcium channel and two ligand-gated ion channels nAChR, without any effect. These toxins are the first described as active on MCRs, as well as the first with this scaffold that do not target ion channels (Reynaud et al., 2020).

Cell-based assay following cAMP expression via the Gαs pathway was used to characterize N-TRTX-Preg1a and N-BUTX-Ptr1a on MC1R and MC4R. On MC1R, both toxins, tested at 100 μM, induced agonistic activity. At the same concentration, the toxins displayed no agonist activity on MC4R, but curiously they were also unable to antagonize MSH activation (Reynaud et al., 2020). Sequence analysis revealed that both toxins share 60% identity with known toxins active on ionic channels. Phylogenic analyses were unable to unambiguously link these melanocortin toxins with their respective group, strongly suggesting that they may form a new sub-group of ICK and CSαβ toxin. The structure of both toxins was solved by NMR with particular attention to the allocation of the disulfide bridges (Figures 4B,C).

## Toxins With 4 Disulfide Bonds

Four-disulfide-bridge toxins (4DBT) are largely present in animal venoms and display an important diversity of structural scaffolds according to their disulfide frameworks. In scorpions, they belong to the cystine-stabilized αβ family with three small antiparallel β-sheets packed against an α-helix, such as in the α-toxins interacting with sodium channels (Bosmans and Tytgat, 2007) or in chlorotoxin, which binds insect chloride channels (Lippens et al., 1995). In spiders, 4DBT mainly belong to the knottin structural family targeting various ion channels, such as the ω-agatoxin IVA interacting with the Ca_V_2 channels (Mintz et al., 1992; Bourinet and Zamponi, 2017) or the µ-agatoxins, which shift voltage-dependent activation of insect Na_V_ channels (Adams, 2004). Cone snails produce in their venoms a huge diversity of toxins, from small linear sequences to large highly reticulated peptides. 4DB conotoxins belong to seven different cysteine framework families (XI, XII, XIII, XV, XVII, XXII, and XXVI) and include several non-natural amino acids (Kaas et al., 2012). The molecular target of these conotoxins is mainly unknown. Finally, in snakes, two different structural families of 4DBT have been identified, the 3_10_β fold of the omwaprin associated with its antimicrobial property (Nair et al., 2007) and the three-finger-fold toxins (3FTs) that often represent the most frequent structures found in *Elapidae* snake venoms. For instance, in the venom gland of the *Dendroaspis angusticeps*, 3FTs represent 70% of all the toxins (Lauridsen et al., 2016), while in the coral snake *Micrurus tschudii* this percentage reaches 95% (Sanz et al., 2016). This fold is characterized by three distinct loops rich in β-sheets emerging from a globular core reticulated by four highly conserved disulfide bridges with C1-C3, C2-C4, C5-C6, and C7-C8 connectivity. Structural variations in the 3FT sequences support the multifunctional properties associated with this scaffold, which can interact with a large diversity of molecular targets. Historically, the first targets identified with 3FTs were the nicotinic acetylcholine receptors (nAChRs), for which these toxins have been used as specific pharmacological and structural tools (Changeux et al., 1970; Kessler et al., 2017). The neurotoxic effect of the *Elapidae* venoms is mainly related to the flaccid paralysis associated with 3FT-muscular nAChR interaction (Chang and Lee, 1963).

3FTs may also inhibit a large diversity of ion channel functions, such as calciseptine, which blocks the L-type Ca-channel (de Weille et al., 1991), δ-calliotoxin, which selectively targets the Na_V_1.4 sodium channel (Yang et al., 2016) or the pain-relieving peptides mambalgins and µ-EPTX-Na1a, which are characterized by their capacity to block acid-sensing ion channels (ASIC) (Diochot et al., 2012), and sodium channel Na_V_1.8 (Zhang et al., 2019), respectively. This structural toxin family is also known to interact with phospholipids to induce cardiotoxic/cytotoxic effects (Konshina et al., 2011) and to abolish enzymatic activities, such as fasciculins on acetylcholinesterase (Bourne et al., 1995) or PLAIγ on PLA2 (Ohkura et al., 1994).

Interestingly, this multifunctional structural scaffold is also able to interact with various GPCR targets belonging to the aminergic receptor family and in particular with muscarinic and adrenergic receptors (Servent and Fruchart-Gaillard, 2009; Servent et al., 2011; Maïga et al., 2012; Näreoja and Näsman, 2012). Furthermore, the former strict classification between muscarinic and adrenergic toxins was challenged by results highlighting the capacity of some toxins to interact simultaneously with high affinity on both types of receptor family. Phylogenic analyses of these toxins show that muscarinic, adrenergic and dopaminergic toxins may be pooled in one family called aminergic toxins, this family coming probably from a specific radiation of ligands present in mamba venoms (Blanchet et al., 2014).

It was from a cDNA library of the king cobra snake (*Ophiophagus hannah*) that β-cardiotoxin was identified before being purified from the venom. Sequence analysis classified this toxin as a cardiotoxin, but only with 55% in sequence identity. Injected in rats, β-cardiotoxins decrease heart rate and induce a negative chronotropic effect, an opposite effect to that of classical cardiotoxins. It was advanced that β-cardiotoxin acts directly on heart muscle. As the β-adrenergic receptors are abundantly expressed in this tissue, the authors performed a binding study on the two β-adrenoceptor subtypes, the β1R and β2R. The toxin has an affinity between 5 and 10 µM for both receptors. Even if no functional assays were performed, we can imagine that β-cardiotoxin has an antagonist effect on β1R (Rajagopalan et al., 2007).

### Muscarinic Toxins: Muscarinic Receptors

Three-finger-fold toxins isolated from mamba venoms are among the more selective mAChR ligands. Over the last 30 years, about 10 different muscarinic toxins (MTs) have been purified and isolated from *Dendroaspis* venoms using a bioguided strategy (Adem et al., 1988; Karlsson et al., 1991; Servent et al., 2011). More recently, proteomic analysis of *Dendroaspis* venoms revealed the overall picture of the toxins present in these venoms and the large proportion of 3FTs, especially in green mambas, suggesting the presence of other muscarinic toxins, not yet identified and sequenced (Laustsen et al., 2015; Lauridsen et al., 2016; Ainsworth et al., 2018).

In addition to these MTs isolated from mamba venoms, some studies have described the presence in other *Elapidae* venoms of muscarinic toxin-like proteins able to display muscarinic activity. For instance, 3FTs with up to 50% sequence identity with MTs have been identified in *Naja kaouthia* venom, but with apparent affinity for mAChRs in the low micromolar range (Kukhtina et al., 2000). More recently, a 3FT with an additional fifth disulfide bridge was isolated from the same venom and shown to induce allosteric modulation on M1, M2, and M3 subtypes at micromolar concentration (Lyukmanova et al., 2015).

Muscarinic receptors (mAChRs) are the metabotropic counterparts of the ionotropic nicotinic receptors (nAChRs), both being activated by acetylcholine (ACh). Molecular cloning has revealed the existence of five distinct mAChR subtypes (M1 to M5), which mediate the action of ACh in almost all tissues, via hormonal and neuronal mechanisms (Eglen, 2012). mAChRs mediate autocrine/paracrine actions of ACh, such as the regulation of cell proliferation and migration, apoptosis, angiogenesis, skin cell signaling, immune functions or cytoskeletal organization. In the central and peripheral nervous systems, the five mAChR subtypes are expressed pre- and post-junctionally with specific distribution. Centrally, they regulate a large number of cognitive, sensory, behavioral or motor functions while at the periphery, via the parasympathetic system, they control glandular secretion, heart rate or smooth muscle contraction (Wess et al., 2007). Even if activation of multiple cellular effector pathways has been described following an agonist-mAChR interaction (Antony et al., 2009), the M1, M3, and M5 subtypes preferentially recruited Gαq/G_11_ activation, leading to an increase in cytosolic Ca^2+^ concentrations, whereas the M2 and M4 subtypes coupled mainly to Gαi/Go-proteins to modulate the activity of adenylyl cyclase and ion channels. As a prototypic class of GPCRs, mAChRs possess orthosteric and allosteric ligand-binding sites, exist in monomeric/dimeric states and in constitutively active forms, or may induce biased signaling, raising interesting opportunities for the design of therapeutic ligands.

Due to their ubiquitous distribution and involvement in several brain, cardiac, digestive and airway functions, disruption of muscarinic signaling contributes to many pathological conditions such as Alzheimer’s disease, Parkinson’s disease or schizophrenia, chronic obstructive pulmonary disease, overactive bladder, cardiovascular diseases and irritable bowel syndrome. A multitude of mAChR ligands have been identified over the years for potential therapeutic applications, but their limited selectivity for one of the five mAChR subtypes has often hampered their clinical development due to adverse side effects (Langmead et al., 2008).

**FIGURE 5:**
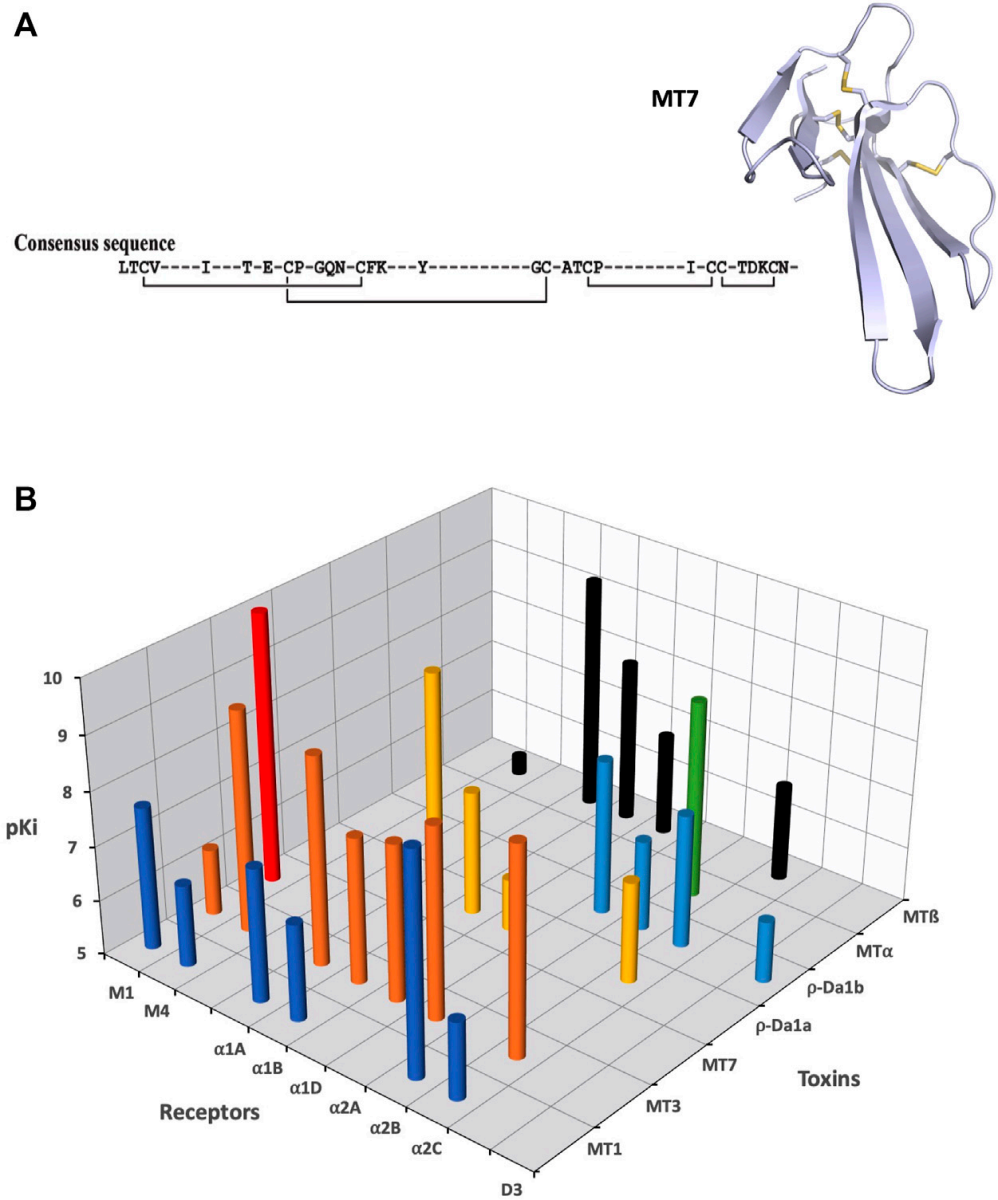
**(A)**, Consensus sequences of the aminergic three-finger fold toxins, with MT7 represented in light grey cartoon with disulfide bonds in yellow. **(B)**, Representation of binding affinities (in pKi) of various toxins for different aminergic receptors.

Three structures of MTs have been solved by NMR (Ségalas et al., 1995) or X-ray crystallography (MT1: 4DO8, MT2: 1FF4 and MT7: 2VLW) (Fruchart-Gaillard et al., 2008, 2012), highlighting that MTs belong to the 3FTs family with all the structural markers of this fold. In addition, MTs include highly conserved sequences at their N-ter (LTCV) region, C-ter region (TDKCN) and in the loops connecting fingers 1–2 (GQN (L/V)CFK) and 2–3 ((A/V)AT) (Figure 5A). Nevertheless, despite high sequence identity (at least 50%), MTs display highly variable pharmacological profiles in term of affinity, selectivity and mode of action (Figure 5B). MT7 is the most potent and selective ligand of the M1 receptor, interacting with sub-nanomolar affinity with this subtype and being unable to recognize the four other mAChRs, even at high micromolar concentration (Max S. et al., 1993; Mourier et al., 2003). Conversely, MT1 and MT3 display polypharmacological profiles, interacting not only with M4 and M1 subtypes (Jolkkonen et al., 1994) (Liang et al., 1996), but also with α1-and α2-adrenergic receptors (Blanchet et al., 2014) (Figure 5B). In addition, in order to specify the mode of action of these toxins on mAChRs, various binding and functional assays have been performed demonstrating that MT7 behaves as an allosteric modulator of M1 functions (Max SI. et al., 1993; Olianas et al., 2000; Fruchart-Gaillard et al., 2006), while MT1 or MT3 are competitive antagonists on M1 and M4 receptors, respectively (Jolkkonen et al., 1995; Olianas et al., 1996; Mourier et al., 2003).

**FIGURE 6:**
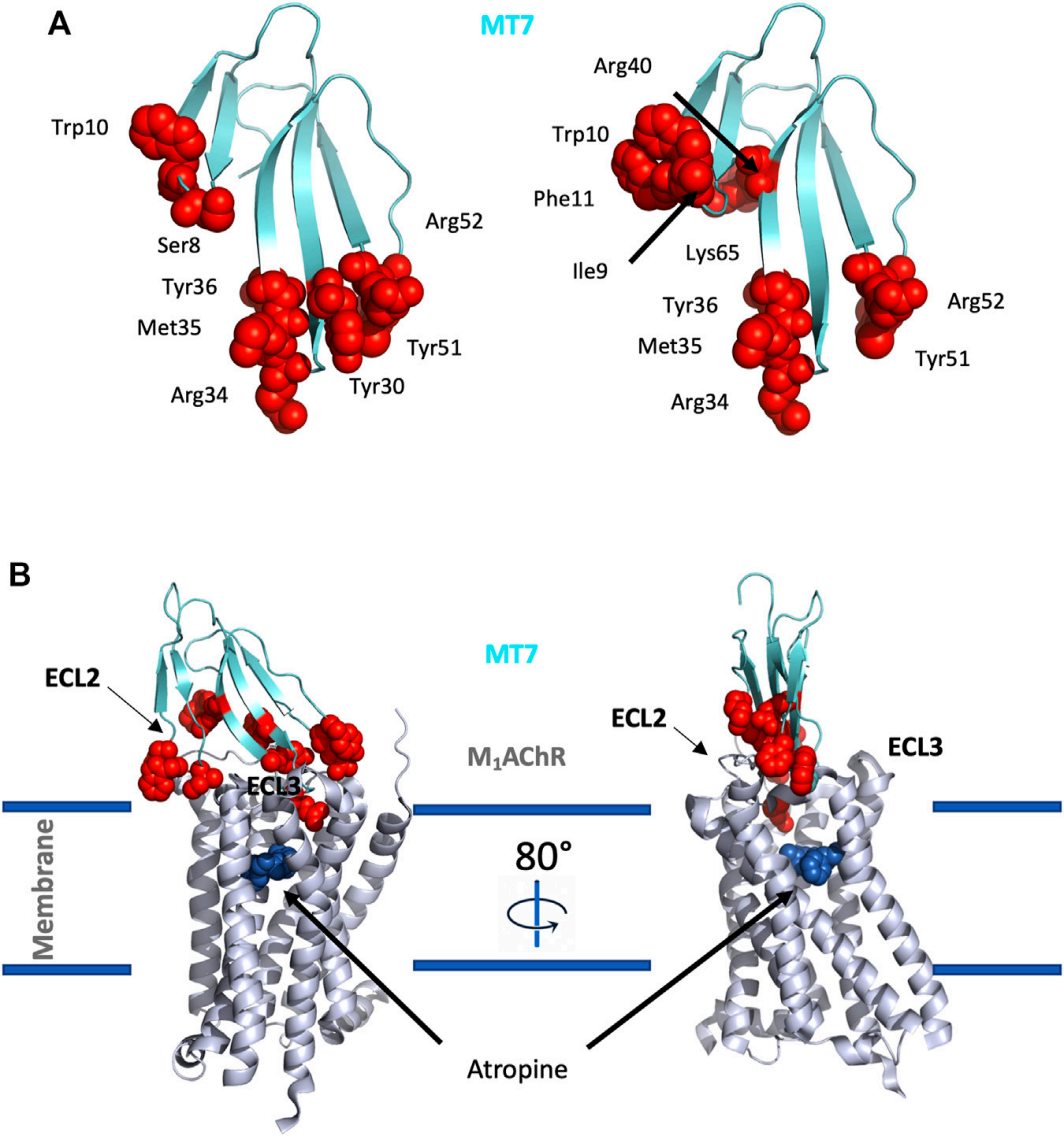
Structure/activity relationship and three-dimensional structure of MT-mAChRs complex. **(A)**, Left: Important residues for binding of MT7 to M1 (2vlw) (Fruchart-Gaillard et al., 2008; Marquer et al., 2011); Right: Structural important residues of MT7 close to M1 (6wjc) (Maeda et al., 2020). Critical residues are shown as red spheres. **(B)**, Structural view of MT7 binding to M1 (Maeda et al., 2020). M1 is shown in light grey cartoon, the antagonist atropine is represented as blue spheres and MT7 is shown as in A.

In order to perform structure-activity relationship studies and identify at the molecular level how MTs interact with mAChRs, the MT7-M1 interaction was selected, based on the high affinity and selectivity of this interaction and on the capacity to produce large quantities (up to 10 mg/synthesis) of wild-type toxin and variants of the toxin by solid-phase peptide synthesis (Mourier et al., 2003). First, alanine-scanning was performed on MT7, highlighting the major role of residues located at the tip of loop I (Trp10), loop II (Arg34, Met35, Tyr36) and loop III (Tyr51, Arg52) in its interaction with the M1 receptor (Fruchart-Gaillard et al., 2008; Marquer et al., 2011) (Figure 6A). Interestingly, these results were confirmed recently with the resolution of the MT7-M1 structural complex where all these residues, and some additional ones (Phe11, Arg40, and Lys65), predominantly contribute to the interaction surface with the receptor (Maeda et al., 2020) (Figure 6B). At the receptor level, Glu170 and Tyr179 of the M1 receptor were first identified as essential for MT7 interaction (Kukkonen et al., 2004), while a complete mutational analysis using chimeric M1-M3 receptors and site-directed mutagenesis approaches revealed the major role of residues in the extracellular loop 2 and top of the TM7 domain of the receptor in the MT7 interaction (Marquer et al., 2011). These preliminary results were recently confirmed, completed and detailed by the resolution of the crystallographic structure of the MT7-hM1 complex (Figure 6B) (Maeda et al., 2020). Maeda and colleagues show that the interactions between M1 mAChR and MT7 occur predominantly with extracellular loop 2 of the receptor, which forms extensive hydrophobic interactions and large polar contacts with MT7’s loop 1 and loop 2, respectively. These interactions and a few others involving receptor transmembrane helices 7 and 4, support the high affinity and selectivity of the MT7-M1 complex (Maeda et al., 2020). Moreover, the authors show that the insertion of finger loop 2 of the toxin into the extracellular vestibule of M1 receptor stabilizes an outward movement of its TM6, extracellular loop 3 and TM7 domains, in agreement with its allosteric property. Finally, based on the structural information coming from this crystallographic complex structure, an *in vitro* engineering of the MT7 loops was performed allowing the selection of an M2-selective ligand, confirming the multipotency of the three-finger scaffold and its ability to support interaction with a large diversity of aminergic GPCR subtypes (Maeda et al., 2020).

*In vivo,* MTs have been used as pharmacological tools to study the functional role of mAChRs in various pathophysiological contexts. For instance, MT3 was shown to be effective at inhibiting the development of myopia and used to demonstrate the existence of different muscarinic mechanisms in excessive eye growth (Nickla et al., 2015). This toxin was also used to show the major role of the M4 subtype in the inhibition of hippocampal and striatal adenylate cyclase activity as well as in the modulation of the neurotransmission of hippocampal neurons (Sánchez, et al., 2009a; Sánchez, et al., 2009b). The exceptional selectivity of MT7 for the M1 subtype was exploited to demonstrate that the potentiation of NMDA receptor currents in hippocampal CA1 pyramidal cells was mediated by the M1 receptor (Marino et al., 1998) or to confirm that the M1 receptor mediates part of nitric oxide’s vagal facilitatory effect in the isolated innervated rat right atrium (Hogan and Markos, 2007). Finally, the group of Fernyhough has shown that, via ERK-CREB signaling activation, MT7 elevates neurite outgrowth and protects from small and large fiber neuropathy in adult sensory neurons (Sabbir and Fernyhough, 2018). This effect is mediated via the enhancement of mitochondrial function and MT7 seems able to prevent neurodegeneration and drive nerve fiber repair in two of the most common forms of peripheral neuropathy, type 1 diabetes and chemotherapy-induced peripheral neuropathy (Saleh et al., 2020).

### ρ-Da1a: α1a-Adrenoceptor

ρ-Da1a (formerly named AdTx-1) was discovered by a bioguided strategy in the venom of the green mamba snake *Dendroaspis angusticeps* by competition with ^3^H-prazosine on rat brain membrane preparations (Quinton et al., 2010). ρ-Da1a displays a high selectivity for α1_A_AR (K_i_ = 0.35 nM) versus the other adrenoceptor subtypes (K_i_ = 317 nM or 420 nM for α1_B_AR and α1_D_AR, respectively) and more broadly as compared to other aminergic receptors. On COS cells stably expressing α1_A_AR, ρ-Da1a acts as a non-competitive antagonist, reducing epinephrine efficacy (Palea et al., 2013). This insurmountable antagonist property was confirmed using four other α1AR agonists (Maïga et al., 2013a). On isolated rat and human prostatic muscles, ρ-Da1a displayed the same insurmountable antagonist properties, making this peptide the most effective relaxant of prostatic muscle and a potential drug candidate for prostate hyperplasia. ρ-Da1a is a 3FT reticulated with four disulfide bridges and belongs to the aminergic-toxin group. It was crystallized and its structure solved by X-ray difraction (Maïga al., 2013). Mutational studies were performed to delineate the mode of action of ρ-Da1a on α1_A_AR. Results described in this review included published (Maïga et al., 2013b) and also original data. The tips of the three-finger toxins are known to interact with their respective targets (Kessler et al., 2017)**.** Variants K7A, I9A, F10A, of the first finger, the K34A and Y36A of the second and the E50A and D53A of the third finger were tested. The Y36A modification in loop II induces a strong decrease in the toxin’s affinity (three order of magnitude) for α1_A_AR, while complementary interactions involved Phe10 in loop I and Asp53 in loop III (Table 1).

**TABLE 1:** Affinities of ρ-Da1a variants on α1_A_AR. Data obtained by competition binding experiments with ^3^H-prazosin on COS cells stably expressing α1_A_AR as previously described (Quinton et al., 2010).

Variants	Ki	pKi	Ratio	N
WT	0.85	9.07 ± 0.03	1	10
K7A	4.5	8.35 ± 0.03**	5.3	3
F10A	15	7.82 ± 0.03***	18	8
K34A	1.1	8.96 ± 0.03	1.3	6
Y36A	1,600	5.80 ± 0.1***	1900	5
E50A	0.2	9.70 ± 0.04	0.24	3
D53A	38	7.42 ± 0.03***	45	4

WT: wild type sequence. Ki are expressed in nM, pKi = −log (Ki), ratio = Ki (variant)/Ki (WT), N: number of determinations. Statistical analysis was done by a one-way ANOVA with *post hoc* test according to Dunnett in comparison to α1_A_AR WT.

**: *p* value = [0.01; 0.001]; ***: *p* value < 0.001 using ANOVA tests. Original data.

We explored the external part of the receptor in addition to the orthosteric site. Curiously, none of the ECL residues are involved in ρ-Da1a binding. Two discontinuous areas were identified, at the top of the TM2 domain with the two residues Phe86 and Glu87, and at the orthosteric site where Phe288 and Phe312 are clearly important for toxin affinity. The aspartate residue in position 3.32 (Asp106 in α1_A_AR) is a key element for the activity of the aminergic agonists. α1_A_AR-D106A was expressed very poorly and binding could be detected only with ^125^I-HEAT. ρ-Da1a affinity is affected by this mutation, demonstrating a contribution of this negative charge in the toxin interaction (Maïga et al., 2013a, 2014) (Table 2).

**TABLE 2:** Affinities of ρ-Da1a on α1_A_AR mutants. Data obtained by competition binding experiments with ^3^H-prazosin and^125^I-HEAT on COS cells stably expressing α1_A_AR as previously described. Positions of amino acids in the transmembrane helices are given according to the Ballesteros-Weinstein nomenclature (Ballesteros and Weinstein, 1995).

α1aAR	Position	pKi using ^3^H-prazosin	pKi using^125^I-HEAT	Ratio
WT		9.19 ± 0.09	9.26 ± 0.07	1
F86A	2.64		7.7 ± 0.06&	36
E87A	2.65	8.14 ± 0.12		11
Y91A	ECL1	9.26 ± 0.08		0.85
F94A	ECL1	9.18 ± 0.14		1.02
R96A	ECL1	9.27 ± 0.11		0.83
F98A	3.24	8.80 ± 0.09		2.45
D106A	3.32		8.48 ± 0.11&	6.0
P168A	ECL2	8,92 ± 0.14		1.86
D172A	ECL2	9.11 ± 0.09		1.20
E173A	ECL2	8.96 ± 0.11		1.70
E180A	ECL2	9.01 ± 0.07		1.51
E181A	ECL2	9.27 ± 0.12		0.83
F187A	5.41	8.96 ± 0.08		1.70
S188A	5.42	9.30 ± 0.13		0.78
S192A	5.46	9.26		0.85
F193A	5.47		9.64 ± 0.11&	0.42
F281A	6.44		9.66 ± 0.09&	0.40
F288A	6.51		8.00 ± 0.08&	18
M292A	6.55		9.41 ± 0.11&	0.71
P299A	ECL3	9.22 ± 0.15		0.93
D300A	ECL3	8.89 ± 0.13		2.00
K302A	ECL3	9.43 ± 0.08		0.58
E305A	ECL3	8.77 ± 0.09		2.63
F308A	7.35		9.15 ± 0.07&	0.71
F312A	7.39		7.28 ± 0.10*	93
W313A	7.40	9.12 ± 0.16		1.17

WT: wild type sequence. Ratio = Ki (variant)/Ki (WT), pKi = −log (Ki). The data are original unless & (Maïga, et al., 2013a). Ratio concern the ρ-Da1a affinity for α1_A_AR mutant *versus* α1_A_AR wild-type.

*: *p* value = [0.0; 0.01] using ANOVA tests.

The potency and selectivity of ρ-Da1a could be useful to clarify the physiological functions of α1_A_AR in animal models. Moreover, this peptide could be the basis for the synthesis of new α1_A_AR antagonists that could be used to treat lower urinary tract symptoms secondary to benign prostatic hyperplasia. The rabbit prostate gland smooth muscle contraction is mainly mediated by activation of α1_A_AR. At concentrations of 30 and 100 nM, ρ-Da1a injected intraperitoneally decreased both phenylephrine potency and efficacy, confirming its *in vivo* insurmountable antagonism (Quinton et al., 2010). The same insurmountable antagonist property was obtained with human isolated prostatic smooth muscle. As expected with the peptide nature of ρ-Da1a, no effect could be seen on the rat intra-urethral pressure when the toxin was administrated *per o*s (Palea et al., 2013). *In vivo* effects of ρ-Da1a and tamsulosine were evaluated in male rat by measuring the intra-urethral pressure. Here again, ρ-Da1a acted as an insurmountable antagonist with a potency equivalent to that of tamsulosin (Palea et al., 2013).

### Comparison MT7/ρ-Da1a

**FIGURE 7:**
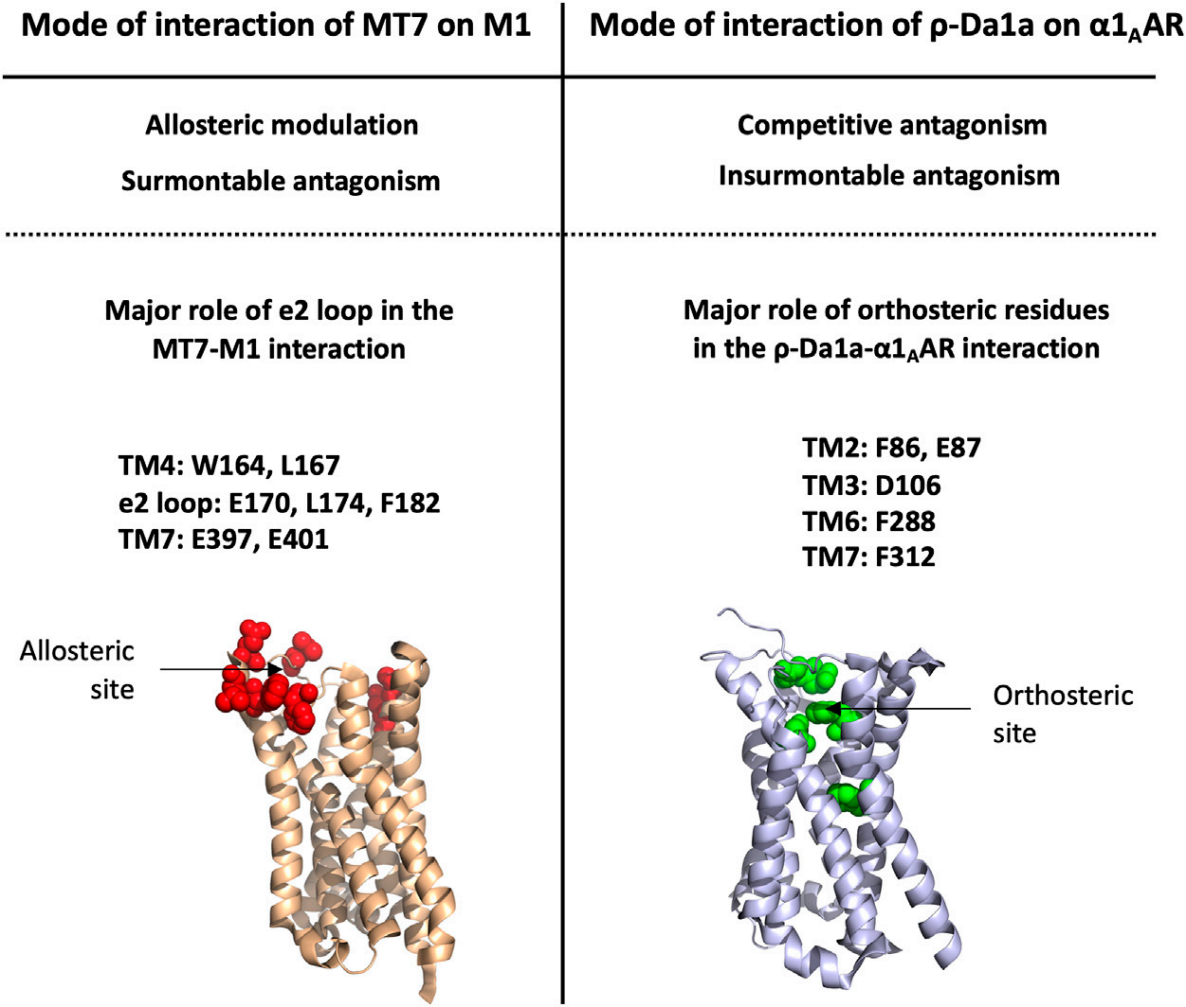
Differences in the mode of interaction of the three-finger fold toxins MT7 and ρ-Da1a with their respective aminergic receptor. Allosteric site of M1 is represented in red, orthosteric site of α1_A_AR is represented in green. Both receptors are shown in the same orientation.

Interestingly, based on the pharmacological and structural characterization of the interaction of aminergic toxins on aminergic receptors described above, the multiplicity of these interactions and the diversity of the biological effects observed were highlighted. Indeed, despite their belonging to the same structural toxin family, the three-finger-fold toxins, and their interaction with phylogenetically related class A aminergic receptors, MT7 and ρ-Da1a interact in a totally different way with their respective receptors and induce quite different functional effects. Whereas MT7 acts as an allosteric modulator on muscarinic M1 receptor by interacting predominantly with its extracellular loop e2 (Glu170, Leu174, and Phe 182) (Maeda et al., 2020), ρ-Da1a behaves as a competitive inhibitor on α1_A_AR, in agreement with the crucial role of orthosteric residues of this receptor in its interaction (Phe86, Phe87 in TM2, Asp106 in TM3, Phe288 in TM6 and Phe312 in TM7) (Table 2) (Figure 7). Concerning the toxins, both MT7 and ρ-Da1a exploit the tip of their three fingers to interact with their respective receptors but interestingly, if the highly conserved positively charged residues at the tip of the second loop of three-finger toxins (Arg34 in MT7) is highly critical in its interaction with M1 receptor, it is not the case for ρ-Da1a (Lys34) on α1_A_AR (Table 1). The crystallographic structure of the MT7-M1 complex allows to precise the hydrophobic and polar interactions involved in this interaction, such precise information being not yet available for the ρ-Da1a-α1_A_AR complex. Furthermore, at the functional level, while ρ-Da1a displays insurmountable antagonism on the agonist activation of α1_A_AR (Maïga et al., 2013b), MT7 produces a surmountable effect on the carbamylcholine-activated M1 receptor (personal data). These data highlight the pleiotropic effect of 3FTs on class A GPCRs, reinforcing their interest as pharmacological tools and original modulators of this receptor family.

## Conclusion

Many venomous animals have evolved venom systems in order to select toxins that interfere with specific physiological systems of prey or predators. Besides the most known and potent neurotoxic and cardiovascular/hemostatic effects associated with respective inhibition of various ion channels or enzymes, other alternative strategies appear to have been selected such as those involving GPCR targets. That is the case of different toxins described in this review, as sarafotoxins from Atractaspis snake venoms that exert their toxic effect by inducing a strong general vasoconstriction leading to heart failure via their interaction with endothelin receptors. In the same way, ρ-TIA conotoxin, a selective α1_A_AR antagonist, induces a striking loss of zebrafish larvae escape that could be crucial for the net hunting strategy of this cone snail. This latter example highlights the importance of using ecologically relevant animal models to decipher the biological role of toxins, and could explain the lack of toxic effect in mammals often associated with GPCR-interacting toxins. Interestingly, when the GPCR-toxin itself is devoid of any toxicity, some authors postulate that a synergistic effect may occur with other toxins present in the venoms, as postulated for muscarinic toxins in Dendroaspis venoms. Nevertheless, in many cases, the question of the functional requirement of GPCR-targeting toxins in venoms is still debated. Anyway, the absence of toxicity of these toxins, associated with usual high affinity, selectivity and stability of these peptides, can be largely exploited for therapeutic development. Indeed, GPCRs represent a family of privileged drug targets covering more than 30% of the drugs approved by the FDA (475 drugs targeting 108 GPCRs) (Hauser et al., 2017). Nevertheless, a general problem associated with GPCR drug discovery research is the lack of new specific ligands of this receptor superfamily. Venom-derived peptides could be part of the solution of this problem, as demonstrated by the anti-diabetic exendin-4 and other molecules in pre-clinical and clinical development.
