# Supplementary material for: Structural and Functional Diversity of Animal Toxins Interacting With GPCRs
Source: Front Mol Biosci. 2022 Feb 7;9:811365. doi: 10.3389/fmolb.2022.811365 (PMC8859281; doi:10.3389/fmolb.2022.811365)
Supplement: Supplementary file 1 [file DataSheet2.PDF]

	Mambaquaretin-1	N-TRTX-Preg1a	N-BUTX-Ptr1a
**Source**	*Dendroaspis angusticeps* (Ciolek et al., 2017)	*Poecilotheria regalis* (Reynaud et al., 2020)	*Parabuthus transvaalicus* (Reynaud et al., 2020)
**Target and pharmacological profile *in vitro***	Antagonist V2R (Ciolek et al., 2017)	Agonist on MC1R (Ki= 1.8 µM), no agonist activity on MC4R (Ki= 7.1 µM) (Reynaud et al., 2020)	Agonist on MC1R (Ki= 2.9 µM), no agonist activity on MC4R (Ki= 2.6 µM) (Reynaud et al., 2020)
**Size and sequence**	57 AA: RPSFCNLPVKPGPCNGFFSAFYYSQKTNKCHSFTYGGCKGN ANRFSTIEKCRRTCVG (Ciolek et al., 2017)	28 AA: RCLHAGAACSGPIQKIPCCTCSRRKCT (Reynaud et al., 2020)	34 AA: QMDMRCSASVECKQKCLKAIGSIFGKCMNKKCKC (Reynaud et al., 2020)
**SAR**	Asn15, Gly16 (Ciolek et al., 2017)		
**Scaffold**	Kunitz-type (Ciolek et al., 2017)	ICK (Reynaud et al., 2020)	CSαβ structure (Reynaud et al., 2020)
**PDB structure**	5M4V (Ciolek et al., 2017)	6saa (Reynaud et al., 2020)	6sab (Reynaud et al., 2020)
***In vivo* effects**	In mice: aquaretic effects (Ciolek et al., 2017)		
**Therapeutical interest**	Polycystic kidney diseases (Ciolek et al., 2017)		

Table S2: Main characteristics of the toxins targeting GPCRs

	ρ-TIA	ρ-Da1a	MT7
**Source**	*Conus tulipa*<br>(Sharpe et al., 2001)	*Dendroaspis angusticeps*<br>(Quinton et al., 2010)	*Dendroaspis angusticeps*<br>(Karlsson et al., 1991)
**Target and pharmacological profile *in vitro***	Antagonist adrenoceptors $\alpha 1_A$AR (IC_{50} =150 µM) , $\alpha 1_B$AR (IC_{50} =70 µM) and $\alpha 1_D$AR (IC_{50} =315 µM)<br>(Sharpe et al., 2001)	Antagonist $\alpha 1_A$AR (K_i = 0.35 nM)<br>(Palea et al., 2013)	Modulator allosteric of M1 (Max et al., 1993a) (Mourier et al., 2003).
**Size and sequence**	19 AA: FNWRCCLIPACRRNHKKFC<br>(Sharpe et al., 2001)	64 AA:<br>LTCVTSKSIFGITTEDCPDGQNLCFKRRHYVVPKIYDSTRGCAATCPIPENYDSHCCKTDKCNE<br>(Quinton et al., 2010)	65 AA:<br>LTCVTSKSIFGITTENCPDGQNLCFKKWYYIVPRYSDITWGCAATCPKPTNVRETIRCCETDKCNE<br>(Fruchart-Gaillard et al., 2008)
**SAR**	<u>Critical residues:</u><br>Asn2, Phe3, Arg4<br>(Ragnarsson et al., 2013)	<u>Critical residues:</u><br>Phe10, Tyr36, Glu50<br>(Maïga et al., 2013; Maïga et al., 2014)	<u>Critical residues:</u><br>Trp10, Arg34, Met35, Tyr36, Tyr51, Arg52<br>(Fruchart-Gaillard et al., 2008; Marquer et al., 2011)
**Scaffold**	2 disulfide bridges α-type toxin, ribbon organization<br>(Sharpe et al., 2001)	4 disulfide bridges, 3FT<br>(Maïga et al., 2013)	4 disulfide bridges, three-finger-fold toxins
**PDB structure**	1ien<br>(Sharpe et al., 2001)	4IYE<br>(Maïga et al., 2013)	2VLW<br>(Fruchart-Gaillard et al., 2012)
***In vivo* effects**	On rat: blocks contractions in the aorta and vas deferens.<br>(Lima et al., 2005)	On isolated rat and human prostatic muscles: relaxant<br>(Maïga et al., 2013)	On the isolated innervated rat right atrium: nitric oxide's vagal facilitatory effect<br>(Hogan & Markos, 2007)
**Therapeutical interest**		Prostate hyperplasia<br>(Palea et al., 2013)	
