# Supplementary material for: Structural and Functional Diversity of Animal Toxins Interacting With GPCRs
Source: Front Mol Biosci. 2022 Feb 7;9:811365. doi: 10.3389/fmolb.2022.811365 (PMC8859281; doi:10.3389/fmolb.2022.811365)
Supplement: Supplementary file 2 [file DataSheet1.PDF]

	Sarafotoxins	Exendin-4	Conopressin-T
**Source**	*Atractaspis engaddensis*<br>(Takasaki et al., 1988; Kloog et al., 1988; Ducancel, 2005)	*Heloderma suspectum*<br>(Eng et al., 1990)	*Conus tulipa*<br>(Dutertre et al., 2008)
**Endogenous ligand**	Endothelins<br>(Ducancel, 2005)	GLP-1<br>(Eng et al., 1990)	Oxytocin, vasopressin<br>(Dutertre et al., 2008)
**Target and pharmacological profile *in vitro***	SRTX-6a: agonist ETB subnM<br>SRTX-6b: agonist ETB and ETA subnM<br>SRTX-6c: agonist ETB subnM<br>(Barton & Yanagisawa, 2019; Ducancel, 2005)	Agonist GLP-1R (IC_{50} = 8.9 nM)<br>(Mann et al., 2010)	Cono-T: antagonist V1aR (Ki = 319 nM), partial agonist OTR and V1bR, no activity on V2R<br>(Dutertre et al., 2008; Dutt et al., 2019)
**Size and sequence**	21 AA: SRTX-6a: CSCKDMTDKECLNFCHQDVIW<br>SRTX-6b: CSCKDMTDKECLYFCHQDVIW<br>SRTX-6c: CTCNDMTDEECLNFCHQDVIW<br>(Takasaki et al., 1988; Kloog et al., 1988)	39 AA: HGEGTFTSDLSKQMEEEAVRLFIEWLKNGGPSSGAPPPS<br>(Eng et al., 1990)	9 AA: CYIQNCLRV<br>(Dutertre et al., 2008)
**SAR**	Critical residues for activity:<br>Trp21, Asp8, Glu10, Phe14<br>Cys1, Cys3, Cys11, Cys15<br>(Nakajima et al., 1989; Tam et al., 1994)	Interaction L-R: (Adelhorst et al., 1994; Suzuki et al., 1989)<br>C-ter of Ex-4 interacts with the N-ter extracellular domain of GLP-1R<br>Positions 7, 10, 12, 13, 15<br>Critical residues for activity: (Runge et al., 2008)<br>His7: stimulating insulin release<br>C-ter PSSGAPPPS = "Trp cage" of Ex-4, absent in GLP-1, not implied in the binding of Ex-4 to GLP-1R but decreases stabilization of GLP-1R<br>Critical residues for agonism/antagonism switch: (Chen et al., 2006; Eng et al., 1992)<br>Gly2-Glu3 → Ser2-Asp3<br>All peptide → Truncated N-ter (1→9)<br>↑ antagonism with Glu16, Val19 and Arg20<br>Critical residues for enhanced half-life: (Doyle et al., 2003)<br>Ala8 → Gly8<br>Additional C-ter extension (9 AA)	Critical residues for agonism/antagonism property:<br>Val9 → antagonist V1aR<br>Critical residues for activity:<br>Arg8: pressor activity<br>Aromatic residue 3: increases selectivity for V1aR and V1bR<br>Basic residue 4 → reduces potency V2R<br>Cono-G *and* Cono-S: additional positive charge in position 4<br>(Dutertre et al., 2008; Giribaldi et al., 2020; Postina et al., 1996)
**Scaffold/PDB structure**	4 disulfide bridges<br>SRTX-6b (5glh)<br>(Shihoya et al., 2018; Shihoya et al., 2016; Izume et al., 2020)	No disulfide bridge, **α**-helix<br>Ex-4(9-39) (3c5t)<br>(Runge et al., 2008)	1 disulfide bridge, cyclic conotoxin<br>(Dutertre et al., 2008)
***In vivo* effects**	On human: oedema, erythema, numbness, general weakness, sweating, pallor, vomiting and watery non-bloody diarrhea<br>(Ducancel, 2005)	On isolated rat islets, inhibits glucagon secretion, stimulates insulin synthesis, protects against ß-cell apoptosis (Silvestre et al., 2003)<br>On patients (T2D) Ex-4 decreases glycaemia and raises the ß-cell sensitivity to glucose (Egan et al., 2003)	Supposed renal homeostasis<br>(Dutertre et al., 2008)
**Therapeutical interest**	IRL-1620 (Sovateltide®): peptidomimetic SRTX, agonist ETB, for acute cerebral ischemic stroke, phase III<br>(Pharmazz, Inc. 2021; Gulati et al., 2021)	Exenatide (Byetta®): antidiabetic (T2D)<br>Exenatide LAR: weekly injection<br>Liraglutide: C-16 free-fatty acid derivative<br>(Drucker et al., 2010)	

Table S1: Main characteristics of the agonist-mimicking toxins

	Contulakin-G	Conorphin-T	MIT1
**Source**	*Conus geographus*<br>(Craig et al., 1999)	*Conus textile*<br>(Luo et al., 2006)	*Dendroaspis polylepis*<br>(Schweitz et al., 1990)
**Endogenous ligand**	Neurotensin<br>(Craig et al., 1999)	Dynorphin-A<br>(Brust et al., 2016)	Prokineticins<br>(Li et al., 2001)
**Target and pharmacological profile *in vitro***	Agonist NTSR1 (IC_{50} = 0.96 µM), NTSR2 (IC_{50} = 0.73 µM), NTSR3 (IC_{50} = 0.25 µM)<br>(Craig et al., 1999)	Agonist KOR (Ki = 80 nM)<br>(Brust et al., 2016)	Agonist PKR1 (Ki = 4.1 nM) and PKR2 (Ki = 0.67 nM)<br>(Masuda et al., 2002)
**Size and sequence**	16 AA: pyroE-SEEGGSNA-[β-D-Galp-(1→3)-α-D-GalpNAc-(1→)-TKKPYIL<br>(Thr10 O-glycosylated) (Craig et al., 1999)	9 AA: NCCRRQICC<br>(Luo et al., 2006)	81 AA;<br>AVITGACERDLQCGKGTCCAVSLWIKSVRVCTPVGTSGEDCHPASHKIPFSGQRMHHTCPCA<br>PNLACVQTSPKKFKCLSKS<br>(Schweitz et al., 1990)
**SAR**	<u>Critical residues:</u><br>C-ter tail PYIL: interaction with NTRS1<br>Uncharged residue 7: decreases desensitization of NTSR1<br>Lys9 → Glu9: decreases activity<br>Thr10 deglycosylated: increases affinity and activity *in vitro*<br>(Lee et al., 2015; Craig et al., 1999)	<u>Critical residues for affinity:</u><br>Asp6 → aromatic: increases affinity<br>Ile7<br><br><u>Critical residues for activity:</u><br>Tyr1 → Asn1: decreases activity<br>Substitution of RRQICC: decreases activity<br>(Brust et al., 2016)	<u>Critical residues for activity:</u><br>AVITGA conserved sequence N-ter tail<br>Cys18 substitution: decreases activity<br><u>Critical residues for agonism/antagonism property:</u><br>Ala1 → Met1<br>Addition Met on N-ter<br>(Boisbouvier et al., 1998; Bullock et al., 2004)
**Scaffold/PDB structure**	Linear<br>Neurotensin<br>(Craig et al., 1999)	2 disulfide bridges<br>(Brust et al., 2016)	10 cysteines<br>(Schweitz et al., 1990)
***In vivo* effects**	On rat: central anti-nociceptive effects, peripheral effects (gastrointestinal motility, vasodilatation)<br>(Craig et al., 1999)	On rat: antinociceptive<br>(Deuis et al., 2015)	On guinea pig: contraction ileum and distal colon<br>(Schweitz et al., 1999)
**Therapeutical interest**	CGX-1160 (synthesized form): orphan drug status for the treatment of chronic intractable pain following intrathecal administration in patients with spinal cord injury by FDA<br>(Sang et al., 2016)	Conorphin-1 (synthetic peptidomimicking): analgesic effects on rat<br>(Deuis et al., 2015)	
